# A new bio-inspired metaheuristic algorithm for solving optimization problems based on walruses behavior

**DOI:** 10.1038/s41598-023-35863-5

**Published:** 2023-05-31

**Authors:** Pavel Trojovský, Mohammad Dehghani

**Affiliations:** Department of Mathematics, Faculty of Science, University of Hradec Králové, Rokitanského 62, Hradec Králové, 500 03 Czech Republic

**Keywords:** Engineering, Mathematics and computing

## Abstract

This paper introduces a new bio-inspired metaheuristic algorithm called Walrus Optimization Algorithm (WaOA), which mimics walrus behaviors in nature. The fundamental inspirations employed in WaOA design are the process of feeding, migrating, escaping, and fighting predators. The WaOA implementation steps are mathematically modeled in three phases exploration, migration, and exploitation. Sixty-eight standard benchmark functions consisting of unimodal, high-dimensional multimodal, fixed-dimensional multimodal, CEC 2015 test suite, and CEC 2017 test suite are employed to evaluate WaOA performance in optimization applications. The optimization results of unimodal functions indicate the exploitation ability of WaOA, the optimization results of multimodal functions indicate the exploration ability of WaOA, and the optimization results of CEC 2015 and CEC 2017 test suites indicate the high ability of WaOA in balancing exploration and exploitation during the search process. The performance of WaOA is compared with the results of ten well-known metaheuristic algorithms. The results of the simulations demonstrate that WaOA, due to its excellent ability to balance exploration and exploitation, and its capacity to deliver superior results for most of the benchmark functions, has exhibited a remarkably competitive and superior performance in contrast to other comparable algorithms. In addition, the use of WaOA in addressing four design engineering issues and twenty-two real-world optimization problems from the CEC 2011 test suite demonstrates the apparent effectiveness of WaOA in real-world applications. The MATLAB codes of WaOA are available in https://uk.mathworks.com/matlabcentral/profile/authors/13903104.

## Introduction

Recently, many optimization problems in science, engineering, industry, and technology must be solved using optimization techniques. From a mathematical point of view, decision variables, constraints, and objective functions are the three main parts of modeling an optimization problem. The purpose of optimization is to quantify the decision variables of the problem so that while respecting the constraints, it leads to achieving the minimum (minimization problems) or maximum (maximization problems) value for the objective function^1^. Applied techniques in solving optimization problems fall into the deterministic and stochastic approaches. To choose the suitable technique to solve an optimization problem, a user needs complete information on comparing problem-solving techniques. In contrast, more than the user's available information is often needed. Stochastic approaches, which are mainly based on random search in the problem-solving space, can deal with black-box problems more simply than many deterministic algorithms. These approaches are also suitable for problems where the evaluations of the functions are corrupted by noise. Each deterministic and stochastic approach has various advantages, and generally, none can be considered superior. More information and a detailed comparison of deterministic and stochastic approaches are provided in Krasov’s book^2^.

As one of the most widely used stochastic approaches, metaheuristic algorithms, using stochastic operators, trial and error concepts, and stochastic search, can provide appropriate solutions to optimization problems without requiring derivative information from the objective function. The simplicity of ideas, easy implementation, independence from the type of problem, and no need for a derivation process, are among the advantages that have led to the popularity and pervasiveness of metaheuristic algorithms among researchers^3^. The optimization process in metaheuristic algorithms begins with the random generation of several initial feasible solutions in the problem search space. Then, in an iterative-based process, based on the effectiveness of the algorithm steps, these initial solutions are improved. Finally, the best solution found during the implementation of the algorithm is introduced as the solution to the problem^4^. However, none of the metaheuristic algorithms guarantee that they will be able to provide the optimal global solution. This insufficiency is due to the nature of random search in these types of optimization approaches. Hence, the solutions derived from metaheuristic algorithms are known as quasi-optimal solutions^5^.

Exploration and exploitation capabilities enable metaheuristic algorithms to provide better quasi-optimal solutions. Exploration refers to the ability to search globally in different areas of the problem-solving space to discover the best optimal area. In contrast, exploitation refers to the ability to search locally around the available solutions and the promising areas to converge to the global optimal. Balancing exploration and exploitation is the key to the success of metaheuristic algorithms in achieving effective solutions^6^. Achieving better quasi-optimal solutions has been the main challenge and reason for researchers' development of various metaheuristic algorithms^7,8^.

The main research question is that despite the numerous metaheuristic algorithms introduced so far, is there still a need to develop new algorithms? The No Free Lunch (NFL) theorem^9^ answers the question that the optimal performance of an algorithm in solving a set of optimization problems gives no guarantee for the similar performance of that algorithm in solving other optimization problems. The NFL theorem concept rejects the hypothesis that a particular metaheuristic algorithm is the best optimizer for all optimization applications over all different algorithms. Instead, the NFL theorem encourages researchers to continue to design newer metaheuristic algorithms to achieve better quasi-optimal solutions for optimization problems. This theorem has also motivated the authors of this paper to develop a new metaheuristic algorithm to address optimization challenges.

This paper’s novelty and contribution are in designing a new metaheuristic algorithm called the Walrus Optimization Algorithm (WaOA), which is based on the simulation of walrus behaviors in nature. The main contributions of this article are as follows:

- The natural behaviors of walruses inspire WaOA's design in feeding when migrating, fleeing, and fighting predators.
- WaOA is mathematically modeled in three phases: exploration, exploitation, and migration.
- The efficiency of WaOA in handling optimization problems is tested on sixty-eight standard objective functions of various types of unimodal, multimodal, the CEC 2015 test suite, and the CEC 2017 test suite.
- WaOA performance is compared with the performance of ten well-known metaheuristic algorithms.
- The success of WaOA in real-world applications is challenged in addressing four engineering design issues and twenty-two real-world optimization problems from the CEC 2011 test suite.

The rest of the paper is as follows. The literature review is presented in the “Literature review” section. The proposed WaOA approach is introduced and modeled in the “Walrus Optimization Algorithm” section. Simulation studies are presented in the “Simulation studies and results” section. The efficiency of WaOA in solving engineering design problems is evaluated in the “WaOA for real world-application” section. Conclusions and future research directions are included in the “Conclusions and future works” section.

## Literature review

Metaheuristic algorithms are based on the inspiration and simulation of various natural phenomena, animal strategies and behaviors, concepts of biological sciences, genetics, physics sciences, human activities, rules of games, and any evolution-based process. Accordingly, from the point of view of the main inspiration used in the design, metaheuristic algorithms fall into five groups: evolutionary-based, swarm-based, physics-based, human-based, and game-based.

Evolutionary-based metaheuristic algorithms have been developed using the concepts of biology, natural selection theory, and random operators such as selection, crossover, and mutation. Genetic Algorithm (GA) is one of the most famous metaheuristic algorithms, which is inspired by the process of reproduction, Darwin's theory of evolution, natural selection, and biological concepts^10^. Differential Evolution (DE) is another evolutionary computation that, in addition to using the concepts of biology, random operators, and natural selection, uses a differential operator to generate new solutions^11^.

Swarm-based metaheuristic algorithms have been developed based on modeling natural phenomena, swarming phenomena, and behaviors of animals, birds, insects, and other living things. Particle Swarm Optimization (PSO) is one of the first introduced metaheuristics methods and was widely used in optimization fields. The main inspiration in designing PSO is the search behaviors of birds and fish to discover food sources^12,13^. Ant Colony Optimization (ACO) is a swarm-based method inspired by the ability and strategy of an ant colony to identify the shortest path between the colony to food sources^14^. Grey Wolf Optimization (GWO) is a metaheuristic algorithm inspired by grey wolves' hierarchical structure and social behavior while hunting^15^. Marine Predator Algorithm (MPA) has been developed inspired by the ocean and sea predator strategies and their Levy flight movements to trap prey^16^. The strategy of the tunicates and their search mechanism in the process of finding food sources and foraging have been the main inspirations in the design of the Tunicate Swarm Algorithm (TSA)^17^. Some other swarm-based methods are White Shark Optimizer (WSO)^18^, Reptile Search Algorithm (RSA)^19^, Raccoon Optimization Algorithm (ROA)^20^, African Vultures Optimization Algorithm (AVOA)^21^, Farmland Fertility Algorithm (FFA)^22^, Slime Mould algorithm (SMA)^23^, Mountain Gazelle Optimizer (MGO)^24^, Sparrow Search Algorithm (SSA)^25^, Whale Optimization Algorithm (WOA)^26^, Artificial Gorilla Troops Optimizer (GTO)^27^, and Pelican Optimization Algorithm (POA)^28^.

Physics-based metaheuristic algorithms have been inspired by physics’ theories, concepts, laws, forces, and phenomena. Simulated Annealing (SA) is one of the most famous physics-based methods, the main inspiration of which is the process of annealing metals. During this physical process, a solid is placed in a heat bath, and the temperature is continuously raised until the solid melts. The solid particles are physically separated or randomly placed. From such a high energy level, the thermal bath cools slowly as the temperature decreases so that the particles can align themselves in a regular crystal lattice structure^29^. Gravitational Search Algorithm (GSA) is a physics-based computational method inspired by the simulation of Newton’s law of universal gravitation and Newton's laws of motion among masses housed in a system^30^. Applying the three concepts of a black hole, white hole, and wormhole in cosmology science has been the inspiration for the design of the Multi-Verse Optimizer (MVO)^31^. Some other physics-based methods are: Water Cycle Algorithm (WCA)^32^, Spring Search Algorithm (SSA)^33^, Atom Search Optimization (ASO)^34^, Quantum-inspired metaheuristic algorithms^35^, Momentum Search Algorithm (MSA)^36^, and Nuclear Reaction Optimization (NRO)^37^.

Human-based metaheuristic algorithms have been developed inspired by human activities, social relationships, and interactions. Teaching Learning Based Optimization (TLBO) is the most widely used human-based metaheuristic algorithm in which the interactions between teacher and students, as well as students with each other in the educational space, are its main source of inspiration^38^. The efforts of two sections of society, including the poor and the rich, to improve their financial situation have been the main idea in the design of Poor and Rich Optimization (PRO)^39^. Some other human-based methods are Archery Algorithm (AA)^40^, Brain Storm Optimization (BSO)^41^, Chef Based Optimization Algorithm (CBOA)^42^, War Strategy Optimization (WSO)^43^, and Teamwork Optimization Algorithm (TOA)^44^.

Game-based metaheuristic algorithms have been introduced based on simulating the rules governing various individual and group games and imitating the behaviors of players, referees, coaches, and other effective interactions. E.g., competition of players in the tug-of-war game under the rules of this game has been the main idea used in designing the Tug-of-War Optimization (TWO) algorithm^45^. Premier Volleyball League (PVL) algorithm is introduced based on mathematical modeling of player interactions, competitions, and coaching instructions during game^46^. Puzzle Optimization Algorithm (POA) is another game-based metaheuristic algorithm that has been produced based on players trying to solve puzzles and getting help from each other to arrange puzzle pieces better^47^. Some other game-based methods are Orientation Search Algorithm (OSA)^48^, Ring Toss Game-Based Optimization (RTGBO)^49^, Football Game Based Optimization (FGBO)^50^, Dice Game Optimization (DGO)^51^, and Orientation Search Algorithm (OSA)^48^.

Based on the best knowledge gained from the literature review, no metaheuristic algorithm has been developed based on the simulation of the behaviors and strategies of walruses. However, intelligent walrus behaviors such as food search, migration, escape, and fighting with predators are prone to designing an optimizer. In the next section, based on the mathematical modeling of natural walrus behaviors, a new metaheuristic algorithm is developed to handle optimization applications to address this research gap.

## Walrus Optimization Algorithm

In this section, employed fundamental inspiration and the theory of the proposed Walrus Optimization Algorithm (WaOA) is stated, then its various steps are modeled mathematically.

### Inspiration of WaOA

**Figure 1:**
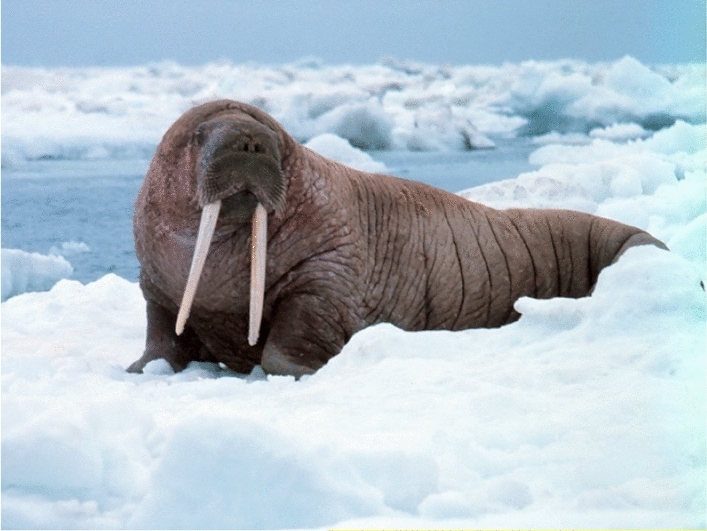
Walrus (the photo is uploaded from Wikimedia^56^).

Walrus is a big flippered marine mammal with a discontinuous distribution in the Arctic Ocean and subarctic waters of the Northern Hemisphere around the North Pole^52^. Adult walruses are easily identifiable with their large whiskers and tusks. Walruses are social animals who spend most of their time on the sea ice, seeking benthic bivalve mollusks to eat. The most prominent feature of walruses is the long tusks of this animal. These are elongated canines seen in both male and female species that may weigh up to 5.4 kg and measure up to 1 m in length. Males' tusks are slightly thicker and longer and are used for dominance, fighting, and display. The most muscular male with the longest tusks dominates the other group members and leads them^53^. An image of walrus is presented in Fig. 1. As the weather warms and the ice melts in late summer, walruses prefer to migrate to outcrops or rocky beaches. These migrations are very dramatic and involve massive aggregations of walruses^54^. The walrus has just two natural predators due to its large size and tusks: the polar bear and the killer whale (orca). Observations show that the battle between a walrus and a polar bear is very long and exhausting, and usually, polar bears withdraw from the fight after injuring the walrus. However, walruses harm polar bears with their tusks during this battle. In the fight against walruses, killer whales can hunt them successfully, with minimal and even no injuries^55^.

The social life and natural behaviors of walruses represent an intelligent process. Of these intelligent behaviors, three are the most obvious:

(i) *Guiding individuals to feed under the guidance of a member with the longest tusks.*

Tracking the best population member in the search process directs the algorithm toward promising areas. In the social life of walruses, the most potent walrus, which can be recognized as having the longest tusk, is responsible for guiding the other walruses. Moving walruses in this process leads to significant changes in their position. Simulating these large displacements increases the algorithm's ability in global search and exploration ability.

(ii) *Migration of walruses to rocky beaches.*

One of the natural behaviors of walruses is their migration due to warming weather in summer. In this process, walruses make big changes in their position by moving towards outcrops or rocky beaches. In the WaOA simulation for a walrus, the position of other walruses are assumed as migration destinations, one of these positions is randomly selected, and the walrus moves towards it. In the design of WaOA, imitating this strategy, global search and discovery capabilities are improved. The difference between the migration strategy and the foraging process under the guidance of the strongest walrus is that in this process, the population update process is prevented from relying on a particular member, such as the best member of the population. This updating process prevents early convergence and the algorithm from getting stuck in local optima.

(iii) *Fight or escape from predators.*

The fighting strategy of walruses in the face of their predators, such as the polar bear and the killer whale, is a long chase process. This chasing process takes place in a small area around the walrus position and causes small changes in the walrus position. Therefore, simulating the small displacements of the walrus by aiming at better positions during the fight leads to an increase in WaOA's ability to search locally and exploit to converge to better solutions.

Mathematical modeling of these behaviors is the primary inspiration for developing the proposed WaOA approach.

### Algorithm initialization

WaOA is a population-based metaheuristic algorithm in which the searcher members of this population are walruses. In WaOA, each walrus represents a candidate solution to the optimization problem. Thus, the position of each walrus in the search space determines the candidate values for the problem variables. Therefore, each walrus is a vector, and the population of walruses can be mathematically modeled using so-called the population matrix. At the beginning of WaOA implementation, populations of walruses are randomly initialized. This WaOA population matrix is determined using (1).

1 $$X = \left[ {\begin{array}{*{20}c} {X_{1} } \\ \vdots \\ {X_{i} } \\ \vdots \\ {X_{N} } \\ \end{array} } \right]_{N \times m} = \left[ {\begin{array}{*{20}l} {x_{1,1} } \hfill & \cdots \hfill & {x_{1,j} } \hfill & \cdots \hfill & {x_{1,m} } \\ \vdots \hfill & \ddots \hfill & \vdots \hfill & {\mathinner{\mkern2mu\raise1pt\hbox{.}\mkern2mu \raise4pt\hbox{.}\mkern2mu\raise7pt\hbox{.}\mkern1mu}} \hfill & \vdots \\ {x_{i,1} } \hfill & \cdots \hfill & {x_{i,j} } \hfill & \cdots \hfill & {x_{i,m} } \\ \vdots \hfill & \ddots \hfill & \vdots \hfill & {\mathinner{\mkern2mu\raise1pt\hbox{.}\mkern2mu \raise4pt\hbox{.}\mkern2mu\raise7pt\hbox{.}\mkern1mu}} \hfill & \vdots \\ {x_{N,1} } \hfill & \cdots \hfill & {x_{N,j} } \hfill & \cdots \hfill & {x_{N,m} } \\ \end{array} } \right]_{N \times m} ,$$

where $X$ is the walruses’ population, ${X}_{i}$ is the $i$th walrus (candidate solution), ${x}_{i,j}$ is the value of the $j$th decision variable suggested by the $i$th walrus, $N$ is the number of walruses, and $m$ is the number of decision variables.

As mentioned, each walrus is a candidate solution to the problem, and based on its suggested values for the decision variables, the objective function of the problem can be evaluated. The estimated values for the objective function obtained from walruses are specified in (2).

2 $$F={\left[\begin{array}{c}{F}_{1}\\ \vdots \\ {F}_{i}\\ \vdots \\ {F}_{N}\end{array}\right]}_{N\times 1}={\left[\begin{array}{c}F\left({X}_{1}\right)\\ \vdots \\ F\left({X}_{i}\right)\\ \vdots \\ F\left({X}_{N}\right)\end{array}\right]}_{N\times 1},$$

where $F$ is the objective function vector and ${F}_{i}$ is the value of the objective function evaluated based on the $i$th walrus.

Objective function values are the best measure of the quality of candidate solutions. The candidate solution that results in the evaluation of the best value for the objective function is known as the best member. On the other hand, the candidate solution that results in the worst value for the objective function is called the worst member. According to the update of the values of the objective function in each iteration, the best and worst members are also updated.

### Mathematical modelling of WaOA

The process of updating the position of walruses in the WaOA is modeled in three different phases based on the natural behaviors of this animal.

#### Phase 1: feeding strategy (exploration)

Walruses have a varied diet, feeding on more than sixty species of marine organisms, such as sea cucumbers, tunicates, soft corals, tube worms, shrimp, and various mollusks^57^. However, walrus prefers benthic bivalve mollusks, particularly clams, for which it forages by grazing around the sea floor, seeking and detecting food with its energetic flipper motions and susceptible vibrissae^58^. In this search process, the strongest walrus with the tallest tusks guides the other walrus in the group to find food. The length of the tusks in the walruses is similar to the quality of the objective function values of the candidate solutions. Therefore, the best candidate solution with the best value for the objective function is considered the strongest walrus in the group. This search behavior of the walruses leads to different scanning areas of the search space, which improves the exploration power of the WaOA in the global search. The process of updating the position of walruses is mathematically modeled based on the feeding mechanism under the guidance of the most vital member of the group, using (3) and (4). In this process, a new position for walrus is first generated according to (3). This new position replaces the previous position if it improves the objective function’s value; this concept is modeled in (4).

3 $${x}_{i,j}^{{P}_{1}}={x}_{i,j}+{rand}_{i,j}\cdot \left({SW}_{j}-{I}_{i,j}\cdot {x}_{i,j}\right)$$

4 $${X}_{i}=\left\{\begin{array}{ll}{X}_{i}^{{P}_{1}}, & {F}_{i}^{{P}_{1}}<{F}_{i},\\ {X}_{i}, & else,\end{array}\right.$$

where ${X}_{i}^{{P}_{1}}$ is the new generated position for the $i$th walrus based on the 1st phase, ${x}_{i,j}^{{P}_{1}}$ is its $j$th dimension, ${F}_{i}^{{P}_{1}}$ is its objective function value, ${rand}_{i,j}$ are random numbers from the interval $\left[0, 1\right]$, $SW$ is the best candidate solution which is considered as the strongest walrus, and ${I}_{i,j}$ are integers selected randomly between 1 or 2. ${I}_{i,j}$ is used to increase the algorithm's exploration ability so that if it is chosen equal to 2, it creates more significant and broader changes in the position of walruses compared to the value of 1, which is the normal state of this displacement. These conditions help improve the algorithm's global search in escaping from the local optima and discovering the original optimal area in the problem-solving space.

#### Phase 2: migration

One of the natural behaviors of walruses is their migration to outcrops or rocky beaches due to the warming of the air in late summer. This migration process is employed in the WaOA to guide the walruses in the search space to discover suitable areas in the search space. This behavioral mechanism is mathematically modeled using (5) and (6). This modeling assumes that each walrus migrates to another walrus (randomly selected) position in another area of the search space. Therefore, the proposed new position is first generated based on (5). Then according to (6), if this new position improves the value of the objective function, it replaces the previous position of walrus.

5 $${x}_{i,j}^{{P}_{2}}=\left\{\begin{array}{c}{x}_{i,j}+{rand}_{i,j}\cdot \left({x}_{k,j}-{I}_{i,j}\cdot {x}_{i,j}\right), {F}_{k}<{F}_{i}\,;\\ {x}_{i,j}+{rand}_{i,j}\cdot \left({x}_{i,j}-{x}_{k,j}\right), else\,,\end{array}\right.$$

6 $${X}_{i}=\left\{\begin{array}{ll}{X}_{i}^{{P}_{2}}, & {F}_{i}^{{P}_{2}}<{F}_{i}\,;\\ {X}_{i}, & else\,,\end{array}\right.$$

where ${X}_{i}^{{P}_{2}}$ is the new generated position for the $i$th walrus based on the 2nd phase, ${x}_{i,j}^{{P}_{2}}$ is its $j$th dimension, ${F}_{i}^{{P}_{2}}$ is its objective function value, ${X}_{k}, k\in \left\{\mathrm{1,2}, \dots ,N\right\} \, \mathrm{and} \, k\ne i,$ is the location of selected walrus to migrate the $i$th walrus towards it, ${x}_{k,j}$ is its $j$th dimension, and ${F}_{k}$ is its objective function value.

#### Phase 3: escaping and fighting against predators (exploitation)

Walruses are always exposed to attacks by the polar bear and the killer whale. The strategy of escaping and fighting these predators leads to a change in the position of the walruses in the vicinity of the position in which they are located. Simulating this natural behavior of walruses improves the WaOA exploitation power in the local search in problem-solving space around candidate solutions. Since this process occurs near the position of each walrus, it is assumed in the WaOA design that this range of walrus position change occurs in a corresponding walrus-centered neighborhood with a certain radius. Considering that in the initial iterations of the algorithm, priority is given to global search in order to discover the optimal area in the search space, the radius of this neighborhood is considered variable so that it is first set at the highest value and then becomes smaller during the iterations of the algorithm. For this reason, local lower/upper bounds have been used in this phase of WaOA to create a variable radius with algorithm repetitions. For simulation of this phenomenon in WaOA, a neighborhood is assumed around each walrus, which first is generated a new position randomly in this neighborhood using (7) and (8). then if the value of the objective function is improved, this new position replaces the previous position according to (9).

7 $${x}_{i,j}^{{P}_{3}}={x}_{i,j}+\left(l{b}_{local,j}^{t}+\left({ub}_{local,j}^{t}-rand\cdot l{b}_{local,j}^{t}\right)\right),$$

8 $$Local \,bounds: \left\{\begin{array}{c}l{b}_{local,j}^{t}=\frac{l{b}_{j}}{t},\\ {ub}_{local,j}^{t}=\frac{u{b}_{j}}{t},\end{array}\right.$$

9 $${X}_{i}=\left\{\begin{array}{ll}{X}_{i}^{{P}_{3}}, & {F}_{i}^{{P}_{3}}<{F}_{i}\,;\\ {X}_{i}, & else\,,\end{array}\right.$$

where ${X}_{i}^{{P}_{3}}$ is the new generated position for the $i$th walrus based on the 3rd phase, ${x}_{i,j}^{{P}_{3}}$ is its $j$th dimension, ${F}_{i}^{{P}_{3}}$ is its objective function value, $t$ is the iteration contour, $l{b}_{j}$ and $u{b}_{j}$ are the lower and upper bounds of the $j$th variable, respectively, $l{b}_{local,j}^{t}$ and ${ub}_{local,j}^{t}$ are local lower and local upper bounds allowable for the $j$th variable, respectively, to simulate local search in the neighborhood of the candidate solutions.

### Repetition process, pseudocode, and flowchart of WaOA

**Figure 2:**
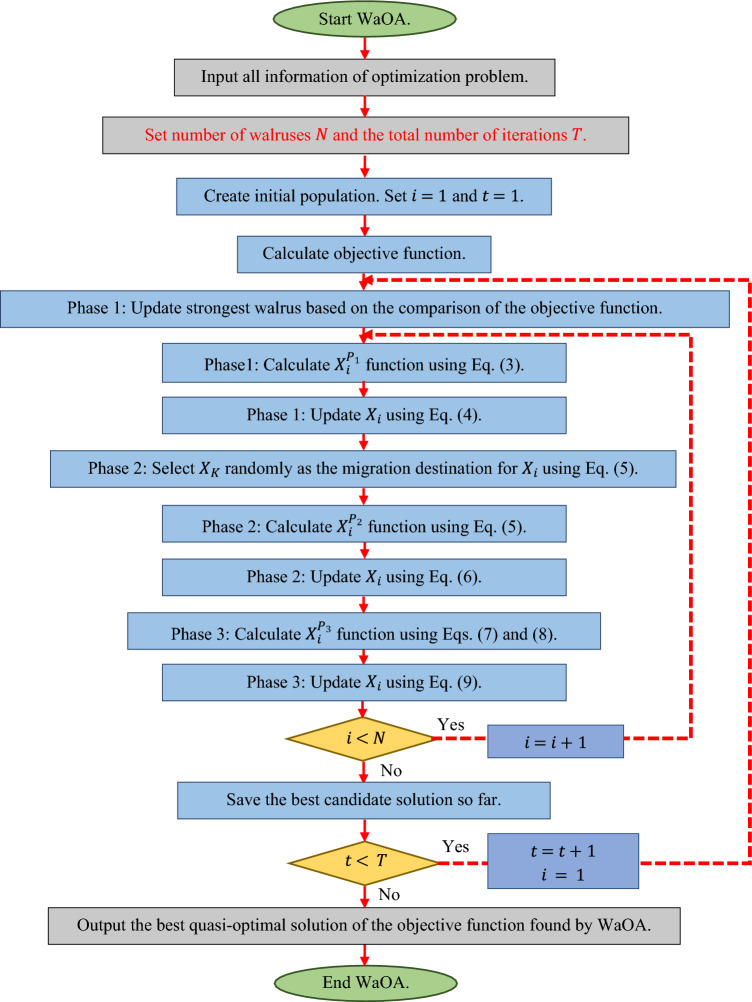
Flowchart of WaOA.


After updating the walruses' position based on the implementation of the first, second, and third phases, the first WaOA iteration is completed, and new values are calculated for the position of the walruses and the objective functions. Update and improve candidate solutions is repeated based on the WaOA steps according to Eqs. (3)–(9) until the final iteration. Upon completion of the algorithm execution, WaOA introduces the best candidate solution found during execution as the solution to the given problem. The WaOA implementation flowchart is presented in Fig. 2, and its pseudocode is specified in Algorithm 1.

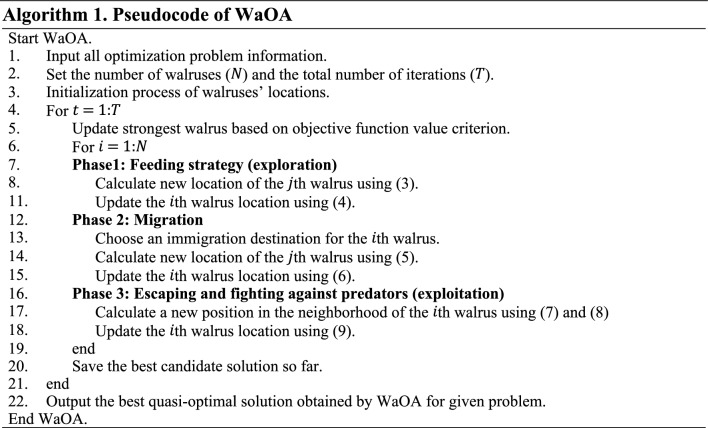


### Computational complexity of WaOA

In this subsection, the computational complexity of WaOA is investigated. WaOA initialization, involving the formation of the population matrix and the calculation of the objective function, has the complexity equal to $O(Nm)$, where *N* is the number of walruses and *m* is the number of problem variables. The WaOA update process has three different phases, each of which has a complexity equal to $O(NmT)$, where *T* is the number of iterations of the algorithm. Thus, the total computational complexity of WaOA is equal to $O(Nm (1 + 3T))$.

Regarding competitor algorithms, GA, PSO, GSA, GWO, MVO, MPA, TSA, RSA, and WSO have a time complexity equal to $O(Nm (1 + T))$, and TLBO has a computational complexity equal to to $O(Nm (1 + 2T))$. Therefore, it is clear that the proposed WaOA approach has higher computational complexity than all algorithms used for comparison. However, to make a fair comparison, we used the population size of each metaheuristic algorithm in the simulation analysis so that the total number of function evaluations is the same for all employed algorithms.

## Simulation studies and results

In this section, WaOA simulation studies on optimization applications are presented. The efficiency of WaOA in providing the optimal solution has been tested on sixty-eight standard objective functions, including unimodal, high-dimensional multimodal, fixed-dimensional multimodal, the CEC 2015 test suite, and the CEC 2017 test suite. The information on these test functions is specified in the Appendix and Tables A1 to A5.

The reasons for choosing these benchmark functions are as follows. Unimodal functions F1 to F7 are suitable for evaluating the exploitation ability of metaheuristic algorithms in convergence towards the global optimal as they do not have a local optimum. Multimodal functions F8 to F23 are suitable options for evaluating the exploration ability of metaheuristic algorithms due to having multiple local optimal. The CEC 2015 and the CEC 2017 test suites have complex benchmark functions that are suitable for evaluating the ability of metaheuristic algorithms to balance exploration and exploitation during the search process. WaOA performance is compared with ten well-known GA, PSO, GSA, TLBO, GWO, MVO, MPA, TSA, RSA, and WSO algorithms to determine the quality of WaOA results. The values set for the control parameters of the employed algorithms are specified in Table 1. The WaOA and mentioned competitor algorithms had been implemented on F1 to F23, each in twenty independent runs containing a thousand iterations (i.e., $T=1000$). In this study, parameter $N$ is considered equal to 20 for WaOA, 30 for TLBO, and 60 for other competitor algorithms to equalize the number of function evaluations. In this case, considering the computational complexity of each algorithm, the number of function evaluations for each metaheuristic algorithm is equal to 60,000.

**Table 1:** Parameter values for the competitor algorithms.

Algorithm	Parameter	Value
WSO	F_min_ and F_max_	0.07, 0.75
	*τ, a*_*o*_*, a*_*1*_*, a*_*2*_	4.125, 6.25, 100, 0.0005
RSA	Sensitive parameter	$\beta =0.01$
	Sensitive parameter	$\alpha =0.1$
	Evolutionary sense (ES)	ES: randomly decreasing values between 2 and − 2
MPA	Constant number	$P = 0.5$
	Random vector	*R* is a vector of uniform random numbers from $\left[0, 1\right]$
	Fish aggregating devices (FADs)	$\mathrm{FADs }= 0.2$
	Binary vector	$U = 0$ or 1
TSA	P_min_ and P_max_	1, 4
	c_*1*_, c_*2,*_ c_*3*_	random numbers lie in the range $\left[0, 1\right]$
MVO	wormhole existence probability (WEP)	Min(WEP) = 0.2 and Max(WEP) = 1
	Exploitation accuracy over the iterations (*p*)	$p = 6$
GWO	Convergence parameter (*a*)	*a*: Linear reduction from 2 to 0
TLBO	${T}_{F}$: teaching factor	${T}_{F} = \mathrm{round} \left[\left(1+rand\right)\right]$
	random number	rand is a random number from $\left[0, 1\right]$
GSA	Alpha, *G*_*0*_, *R*_norm_, *R*_powe*r*_	20, 100, 2, 1
PSO	Topology	Fully connected
	Cognitive and social constant	(c_*1*_, c_*2*_$$)=(2, 2)$$
	Inertia weight	Linear reduction from 0.9 to 0.1
	Velocity limit	10% of dimension range
GA	Type	Real coded
	Selection	Roulette wheel (Proportionate)
	Crossover	Whole arithmetic ($\mathrm{Probability} = 0.8$, $\alpha \in \left[-0.5, 1.5\right]$)
	Mutation	Gaussian ($\mathrm{Probability} = 0.05$)

Optimization results are reported using four statistical indicators: mean, best, standard deviation, and median. In addition, each algorithm's rank in handling each objective function is determined based on the average criterion.

### Evaluation unimodal objective function

Unimodal objective functions have been selected to evaluate the WaOA exploitation ability in local search due to having only one main optimal solution and thus lacking local solutions. The results of optimizing the F1 to F7 functions using WaOA and competitor algorithms are released in Table 2. The simulation results show that WaOA has made the optimal global solution available for the F1, F3, F5, and F6 objective functions. WaOA is also the best optimizer for optimizing F2, F4, and F7. A comparison of optimization results shows that WaOA has a very competitive and obvious superiority over the ten compared algorithms.

**Table 2:** Results of optimization of WaOA and competitor metaheuristics on unimodal functions.

	GA	PSO	GSA	TLBO	GWO	MVO	TSA	MPA	RSA	WSO	WaOA
F_1_											
Avg	35.636479	0.2070031	1.07E−16	1.65E−74	5.55E−59	0.1484659	2.80E−50	5.38E−48	2.361E−74	139.30155	0
Std	15.366825	0.8680742	5.21E−17	4.00E−74	8.50E−59	0.0286353	4.26E−50	9.65E−48	1.056E−73	159.25892	0
Bsf	15.574823	9.38E−05	4.92E−17	1.87E−76	2.00E−61	0.0877539	1.20E−52	2.42E−50	6.72E−275	24.516281	0
Med	32.554042	0.0035476	9.50E−17	2.54E−75	1.52E−59	0.1522978	1.03E−50	1.43E−48	7.55E−150	63.741662	0
Rank	10	9	7	2	4	8	5	6	3	11	1
F_2_											
Avg	2.9137603	1.083336	5.42E−08	7.38E−39	9.23E−35	0.2630586	4.05E−28	1.53E−28	1.545E−57	1.378263	1.02E−294
Std	0.4876288	0.9126523	9.52E−09	6.86E−39	8.85E−35	0.0465101	5.46E−28	2.55E−28	6.908E−57	0.670443	0
Bsf	1.8464391	0.213643	4.10E−08	7.03E−40	1.07E−35	0.1766104	3.46E−30	2.84E−31	6.05E−302	0.5153244	1.93E−301
Med	2.8264357	0.7554828	5.44E−08	4.86E−39	6.07E−35	0.2675488	1.61E−28	3.04E−29	1.62E−171	1.1488451	1.04E−296
Rank	11	9	7	3	4	8	6	5	2	10	1
F_3_											
Avg	2280.3282	810.43051	429.96791	1.92E−25	7.28E−15	14.567466	2.04E−12	8.18E−10	21.200999	1736.8697	0
Std	537.7821	1485.5826	143.79514	2.64E−25	2.78E−14	7.4619678	5.45E−12	3.57E−09	94.81375	884.82296	0
Bsf	1580.7695	28.031944	134.69855	1.95E−28	4.91E−20	3.8558441	1.41E−21	3.67E−19	1.66E−286	456.77681	0
Med	2185.7918	311.51047	404.69921	8.93E−26	8.80E−17	13.32036	1.21E−13	2.62E−14	5.19E−159	1405.5958	0
Rank	11	9	8	2	3	6	4	5	7	10	1
F_4_											
Avg	3.1939824	6.0450436	1.2460337	2.88E−30	1.16E−14	0.5305654	2.22E−19	0.0128207	6.3E−86	17.636532	2.12E−277
Std	0.6363611	2.1930237	1.20872	5.58E−30	1.63E−14	0.2165466	1.90E−19	0.0236447	2.817E−85	3.8176138	0
Bsf	2.1069528	2.8440653	1.17E−08	3.06E−31	4.22E−16	0.1732463	1.07E−20	1.28E−05	1.46E−292	10.597995	2.33E−283
Med	3.1281948	5.5628555	0.8672083	1.01E−30	4.90E−15	0.5082758	1.74E−19	0.0014648	2.48E−179	17.27578	4.21E−280
Rank	9	10	8	3	5	7	4	6	2	11	1
F_5_											
Avg	447.41975	9081.0534	26.444544	26.879052	26.647637	210.87972	23.770128	28.627328	11.588225	8219.6542	0
Std	128.52693	27,683.869	1.2663773	0.9415733	0.6191676	604.31395	0.6101773	0.4200296	14.561344	15,186.046	0
Bsf	266.32221	25.105404	23.188877	25.707118	26.032245	27.165606	23.003447	27.953193	1.673E−28	804.09074	0
Med	446.1339	84.612956	26.332793	26.414859	26.247344	29.66712	23.695067	28.82189	1.935E−26	2420.2388	0
Rank	9	11	4	6	5	8	3	7	2	10	1
F_6_											
Avg	31.42673	0.0510473	9.32E−17	1.1573232	0.6692467	0.1400393	1.61E−09	3.9030313	6.3563416	78.328797	0
Std	10.245634	0.1096987	3.76E−17	0.308507	0.3126824	0.0321654	6.46E−10	0.652961	1.3479915	64.058682	0
Bsf	12.842622	0.0001209	4.17E−17	0.5427727	0.2497602	0.0819428	8.09E−10	3.0609791	3.0286991	23.315452	0
Med	31.320071	0.0086778	8.77E−17	1.1353832	0.7321052	0.1430209	1.37E−09	3.8124693	7.0490774	50.668202	0
Rank	10	4	2	7	6	5	3	8	9	11	1
F_7_											
Avg	0.0092806	0.1547606	0.0469892	0.0018942	0.0008859	0.0115807	0.0006818	0.0037947	7.607E−05	5.67E−05	1.43E−05
Std	0.0032501	0.0722997	0.0156823	0.0017094	0.0004986	0.0048849	0.0004792	0.0018803	5.737E−05	5.778E−05	1.15E−05
Bsf	0.0033147	0.0767143	0.0226519	0.000295	0.0003704	0.0048829	9.71E−05	0.0011774	1.747E−06	3.918E−07	3.75E−07
Med	0.0090163	0.1167272	0.0442485	0.0013531	0.0006481	0.0114417	0.000617	0.0035579	6.982E−05	3.295E−05	1.20E−05
Rank	8	11	10	6	5	9	4	7	3	2	1
Sum rank	68	63	46	29	32	51	29	44	28	65	7
Mean rank	9.714286	9	6.571429	4.142857	4.571429	7.285714	4.142857	6.285714	4	9.285714	1
Total rank	10	8	6	3	4	7	3	5	2	9	1

### Evaluation high-dimensional multimodal objective functions

High dimensional multimodal functions with several local and globally optimal solutions have been selected to evaluate WaOA exploration capability in global search. The optimization results of F8 to F13 functions using WaOA and competitor algorithms are reported in Table 3. What can be deduced from the results of this table is that WaOA has converged to the global optimal in optimizing F9 and F11. WaOA is also the best optimizer for optimizing F10, F12, and F13. TSA is the best optimizer for the F8 objective function, while WaOA is the second-best optimizer for this objective function. Analysis of the simulation results shows that WaOA has an acceptable performance in optimizing high-dimensional multimodal objective functions and has provided a superior outcome compared to ten competitor algorithms.

**Table 3:** Results of optimization of WaOA and competitor metaheuristics on the high-dimensional multimodal functions.

	GA	PSO	GSA	TLBO	GWO	MVO	TSA	MPA	RSA	WSO	WaOA
F_8_											
Avg	− 8732.0566	− 6655.931	− 2500.7139	− 5231.075	− 6083.0982	− 7816.9559	− 9767.2623	− 6168.7082	− 5406.0627	− 7093.1044	− 8881.1061
Std	599.35054	796.53218	393.55973	587.7787	1045.1408	651.41296	475.49897	492.70948	338.83459	1097.2857	152.94837
Bsf	− 9653.3571	− 7989.6611	− 3246.4966	− 6299.8836	− 7654.2152	− 9073.2252	− 10,689.627	− 7227.6685	− 5655.4772	− 9790.4976	− 9075.5449
Med	− 8768.4313	− 6498.1012	− 2466.9839	− 5134.4807	− 6019.692	− 7735.8964	− 9758.4183	− 6271.1354	− 5493.351	− 7050.2823	− 8917.6187
Rank	3	6	11	10	8	4	1	7	9	5	2
F_9_											
Avg	57.122334	62.018927	25.868921	0	0.1072815	95.254712	0	166.51009	0	27.043749	0
Std	15.547508	14.674411	6.6313076	0	0.4797776	24.107803	0	41.096443	0	6.1049913	0
Bsf	34.598517	44.773094	9.9495906	0	0	43.910246	0	91.619574	0	15.188582	0
Med	54.083175	56.224581	26.863884	0	0	91.11852	0	171.11072	0	26.372688	0
Rank	5	6	3	1	2	7	1	8	1	4	1
F_10_											
Avg	3.5854087	3.2364098	8.30E−09	4.09E−15	1.67E−14	0.5370924	3.91E−15	1.7230212	8.882E−16	4.7802266	2.13E−15
Std	0.4151563	0.8973082	1.60E−09	1.09E−15	3.15E−15	0.6513908	1.30E−15	1.6183189	0	0.8814042	1.74E−15
Bsf	2.856219	1.7750878	5.13E−09	8.88E−16	1.15E−14	0.0918005	8.88E−16	1.51E-14	8.882E−16	2.8691584	8.88E−16
Med	3.5140585	3.1264706	8.16E−09	4.44E−15	1.51E−14	0.1269169	4.44E−15	2.7111539	8.882E−16	4.8412315	8.88E−16
Rank	10	9	6	4	5	7	3	8	1	11	2
F_11_											
Avg	1.5658187	0.0989545	8.7022139	0	0.0032973	0.4116334	0	0.0065336	0	2.0387294	0
Std	0.1869219	0.1284744	4.6881208	0	0.0062632	0.1070168	0	0.0063468	0	1.7243749	0
Bsf	1.2168372	0.0001701	2.7594413	0	0	0.2741324	0	0	0	1.107167	0
Med	1.5528338	0.0628106	8.2290232	0	0	0.3958958	0	0.0088858	0	1.5129228	0
Rank	6	4	8	1	2	5	1	3	1	7	1
F_12_											
Avg	0.154488	1.5947308	0.3648692	0.0821009	0.0413696	1.460569	1.82E−10	7.0583987	1.3120904	2.8972544	1.57E−32
Std	0.1110493	1.3141826	0.5614192	0.0268307	0.0193412	1.458994	9.87E−11	3.6829525	0.3309185	1.2781011	2.81E−48
Bsf	0.0487615	0.0004665	3.54E−19	0.0454024	0.0134185	0.0019963	4.63E−11	0.5684754	0.6975687	0.6649101	1.57E−32
Med	0.1255449	1.4939335	0.132149	0.0812882	0.0370402	1.0833741	1.53E−10	6.8697136	1.5217562	2.8024353	1.57E−32
Rank	5	9	6	4	3	8	2	11	7	10	1
F_13_											
Avg	2.2803846	5.1857653	0.2491467	1.0496147	0.5714104	0.0242273	0.0013037	2.8069554	5.442E−22	8081.2485	1.35E−32
Std	0.9391074	4.2390635	0.7537801	0.2541193	0.2372261	0.0208494	0.0038377	0.5753073	2.344E−21	23,135.549	2.81E−48
Bsf	0.9745414	0.2328254	5.86E−18	0.5896332	0.1002619	0.00478	6.66E−10	1.3560246	1.059E−31	13.356494	1.35E−32
Med	2.0472332	4.7415948	1.19E−17	1.0475961	0.6576587	0.0175472	3.03E−09	2.8573762	7.794E−31	38.79782	1.35E−32
Rank	8	10	5	7	6	4	3	9	2	11	1
Sum rank	37	44	39	27	26	35	11	46	21	48	8
Mean rank	6.16667	7.33333	6.5	4.5	4.33333	5.83333	1.83333	7.66667	3.5	8	1.33333
Total rank	7	9	8	5	4	6	2	10	3	11	1

### Evaluation fixed-dimensional multimodal objective function

The fixed-dimensional multimodal functions, which have fewer local solutions than functions F8 to F13, have been selected to evaluate WaOA's ability to balance exploration and exploitation. The optimization results of F14 to F23 functions are reported in Table 4. The results show that WaOA ranks first as the best optimizer in handling all F14 to F23 functions. Furthermore, analysis of the simulation results shows the superiority of WaOA over ten compared algorithms due to the high power of WaOA in balancing exploration and exploitation.

**Table 4:** Results of optimization of the WaOA and competitor metaheuristics on fixed-dimensional multimodal functions.

	GA	PSO	GSA	TLBO	GWO	MVO	TSA	MPA	RSA	WSO	WaOA
F_14_											
Avg	0.9981643	2.5666387	3.5074504	1.2956191	4.5112709	0.9980038	0.9980038	9.8626047	3.4763804	1.0972089	0.9980038
Std	0.0004209	3.1924333	2.2232827	0.7268706	4.9191731	5.84E−12	7.20E−17	4.4088461	2.5684321	0.4436585	1.02E−16
Bsf	0.9980038	0.9980038	0.9980038	0.9980038	0.9980038	0.9980038	0.9980038	0.9980038	1.0478432	0.9980038	0.9980038
Med	0.998005	1.9920309	2.4246779	0.9980039	0.9980038	0.9980038	0.9980038	10.763181	2.9821052	0.9980038	0.9980038
Rank	3	6	8	5	9	2	1	10	7	4	1
F_15_											
Avg	0.0088845	0.0025359	0.0038451	0.0014762	0.0053342	0.0046946	0.0003075	0.006305	0.001262	0.0013103	0.0003075
Std	0.0086194	0.0061158	0.0030393	0.0044582	0.0089026	0.0080421	3.92E−19	0.0138809	0.0005632	0.0044846	9.87E−20
Bsf	0.0008345	0.0003075	0.0013735	0.0003122	0.0003075	0.0003268	0.0003075	0.0003076	0.0006652	0.0003075	0.0003075
Med	0.0051832	0.0003075	0.002212	0.0003187	0.0003079	0.0007413	0.0003075	0.0004825	0.0011743	0.0003075	0.0003075
Rank	11	6	7	5	9	8	2	10	3	4	1
F_16_											
Avg	− 1.0316252	− 1.0316285	− 1.0316285	− 1.0316268	− 1.0316284	− 1.0316284	− 1.0316285	− 1.0284655	− 1.0295585	− 1.0316284	− 1.0316285
Std	9.20E−06	1.61E−16	1.02E−16	1.46E−06	4.11E−09	4.04E−08	2.10E−16	0.0097351	0.0069817	3.273E−08	2.28E−16
Bsf	− 1.0316285	− 1.0316285	− 1.0316285	− 1.0316284	− 1.0316285	− 1.0316285	− 1.0316285	− 1.0316284	− 1.0316235	− 1.0316285	− 1.0316285
Med	− 1.0316282	− 1.0316285	− 1.0316285	− 1.0316273	− 1.0316284	− 1.0316284	− 1.0316285	− 1.0316283	− 1.0312763	− 1.0316285	− 1.0316285
Rank	6	1	1	5	2	4	1	8	7	3	1
F_17_											
Avg	0.3980165	0.6018112	0.3978874	0.3980571	0.3979973	0.3978874	0.3978874	0.3979132	0.4116021	0.3978874	0.3978874
Std	0.0003531	0.5653864	0	0.0002036	0.0004897	1.30E−07	0	3.52E−05	0.0206876	0	0
Bsf	0.3978874	0.3978874	0.3978874	0.3978876	0.3978874	0.3978874	0.3978874	0.3978879	0.3979635	0.3978874	0.3978874
Med	0.3978938	0.3978874	0.3978874	0.3980127	0.3978875	0.3978874	0.3978874	0.3979045	0.4031937	0.3978874	0.3978874
Rank	5	8	1	6	4	2	1	3	7	1	1
F_18_											
Avg	3.0098143	3	3	3.0000008	3.0000125	3.0000004	3	8.8017182	7.5115846	3	3
Std	0.0243289	2.87E−15	3.44E−15	1.14E−06	1.28E−05	3.31E−07	1.23E−15	20.497606	11.136857	3.529E−16	5.76E−16
Bsf	3.0000007	3	3	3	3.0000001	3	3	3.0000003	3.0000033	3	3
Med	3.0001376	3	3	3.0000007	3.0000086	3.0000003	3	3.0000084	3.0001994	3	3
Rank	8	3	4	6	7	5	2	10	9	1	1
F_19_											
Avg	− 3.8626818	− 3.8241312	− 3.8627821	− 3.8617086	− 3.8612086	− 3.862782	− 3.8627821	− 3.8627425	− 3.8195154	− 3.8627821	− 3.8627821
Std	0.0002087	0.1728521	1.97E−15	0.0023471	0.0028936	1.67E−07	2.28E−15	2.60E−05	0.0360682	2.278E−15	2.28E−15
Bsf	− 3.8627821	− 3.8627821	− 3.8627821	− 3.862751	− 3.8627816	− 3.8627821	− 3.8627821	− 3.862781	− 3.8621529	− 3.8627821	− 3.8627821
Med	− 3.8627639	− 3.8627821	− 3.8627821	− 3.8625048	− 3.8627639	− 3.8627821	− 3.8627821	− 3.8627476	− 3.825845	− 3.8627821	− 3.8627821
Rank	4	7	1	5	6	2	1	3	8	1	1
F_20_											
Avg	− 3.2074926	− 3.2857259	− 3.3219952	− 3.2448865	-3.26319	− 3.2564435	− 3.3219952	− 3.2610207	− 2.5357686	− 3.3160412	− 3.3219952
Std	0.1330767	0.0665186	3.81E−16	0.0681168	0.0698817	0.0608337	4.44E−16	0.08834	0.4743104	0.0265835	4.44E−16
Bsf	− 3.3201329	− 3.3219952	− 3.3219952	− 3.3165345	− 3.3219943	− 3.321995	− 3.3219952	− 3.3216262	− 3.0036949	− 3.3219952	− 3.3219952
Med	− 3.2347512	− 3.3219952	− 3.3219952	− 3.2495147	− 3.321992	− 3.2030757	− 3.3219952	− 3.3201103	− 2.7683014	− 3.3219952	− 3.3219952
Rank	8	3	1	7	4	6	1	5	9	2	1
F_21_											
Avg	− 5.1961582	− 4.5268585	− 6.3750274	− 6.231103	− 9.900112	− 7.6132836	− 10.1532	− 7.4645915	− 5.055196	− 8.4065104	− 10.1532
Std	2.5341696	3.0135558	3.5951183	1.9403488	1.1297318	2.6059	2.08E−15	3.1774824	2.788E−07	3.1433508	3.21E−15
Bsf	− 9.8099505	− 10.1532	− 10.1532	− 9.2118711	− 10.153084	− 10.153189	− 10.1532	− 10.099531	− 5.0551966	− 10.1532	− 10.1532
Med	− 5.2428461	− 2.6828604	− 5.3837395	− 6.902018	− 10.152715	− 7.6269292	− 10.1532	− 9.8476972	− 5.0551959	− 10.1532	− 10.1532
Rank	8	10	6	7	2	4	1	5	9	3	1
F_22_											
Avg	− 5.8434567	− 6.4347072	− 10.402941	− 7.3995435	− 10.402536	− 9.605593	− 10.402941	− 4.6611193	− 5.0876679	− 9.3525262	− 10.402941
Std	2.7273583	3.7465155	4.08E−15	2.1676383	0.0001684	1.947217	3.65E−15	3.2949226	9.805E−07	2.5719025	3.05E−15
Bsf	− 10.345307	− 10.402941	− 10.402941	− 10.0663	− 10.402846	− 10.402934	− 10.402941	− 10.354892	− 5.0876699	− 10.402941	− 10.402941
Med	− 5.1988266	− 5.1082473	− 10.402941	− 7.8935553	− 10.402537	− 10.402859	− 10.402941	− 2.7559742	− 5.0876678	− 10.402941	− 10.402941
Rank	7	6	1	5	2	3	1	9	8	4	1
F_23_											
Avg	− 7.3214569	− 7.1817094	− 10.53641	− 8.0026439	− 10.535988	− 9.1867389	− 10.53641	− 6.9534073	− 5.1284729	− 8.7165214	− 10.53641
Std	2.583081	3.8817584	1.68E−15	2.127303	0.0002421	2.3983439	2.51E−15	3.6225412	1.283E−06	3.2430933	1.82E−15
Bsf	− 10.20802	− 10.53641	− 10.53641	− 10.444176	− 10.536352	− 10.536387	− 10.53641	− 10.422351	− 5.128476	− 10.53641	− 10.53641
Med	− 8.5115441	− 10.53641	− 10.53641	− 8.8302784	− 10.536025	− 10.536346	− 10.53641	− 7.7068006	− 5.1284726	− 10.53641	− 10.53641
Rank	7	8	2	6	3	4	2	9	10	5	1
Sum rank	67	58	32	57	48	40	13	72	77	28	10
Mean rank	6.7	5.8	3.2	5.7	4.8	4	1.3	7.2	7.7	2.8	1
Total rank	9	8	4	7	6	5	2	10	11	3	1

**Figure 3:**
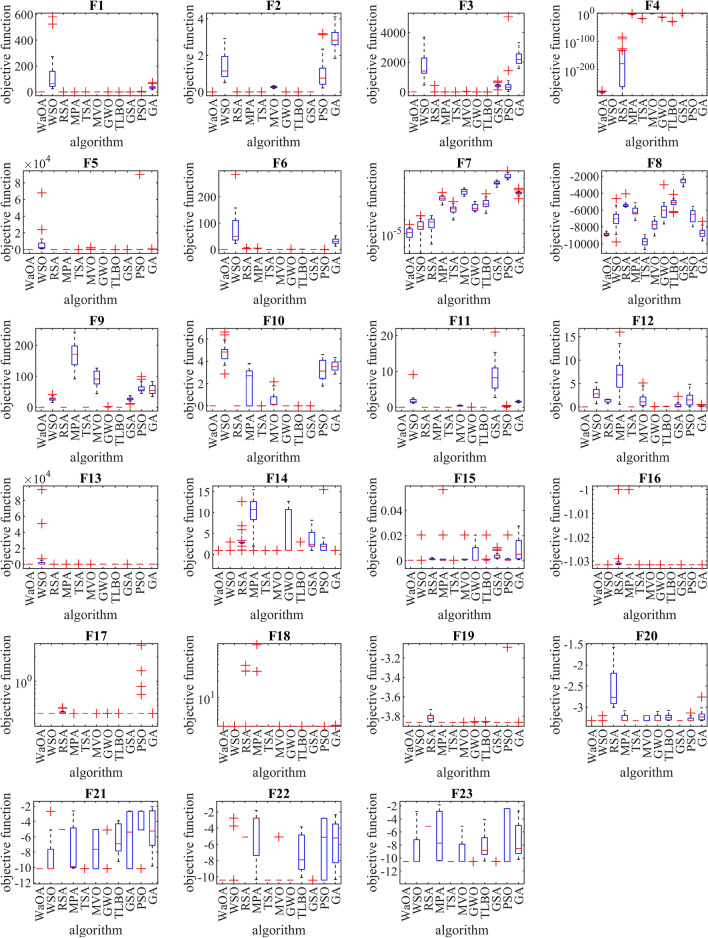
The boxplot diagram of WaOA and competitor algorithms performances on functions F1 to F23.

The performances of WaOA and competitor algorithms in solving F1 to F23 functions are presented as boxplot diagrams in Fig. 3. Intuitive analysis of these boxplots shows that the proposed WaOA approach has provided superior and more effective performance than competitor algorithms by providing better results in statistical indicators in most of the benchmark functions.

### Statistical analysis

In this subsection, the superiority of WaOA over competitor algorithms is statistically analyzed to determine whether this superiority is significant or not. To perform statistical analysis on the obtained results, Wilcoxon signed-rank test^59^ is utilized. Wilcoxon signed-rank test is a non-parametric test that is used to detect significant differences between two data samples. The results of statistical analysis using this test are presented in Table 5. What can be seen from the study of the simulation results is that WaOA has a significant statistical superiority over the competitor algorithm in cases where the $p$-value is less than 0.05.

**Table 5:** Results of Wilcoxon signed-rank test.

Compared algorithms	Functions type		
	Unimodal	High-multimodal	Fixed-multimodal
WaOA vs. WSO	1.01E−24	6.25E−18	1.44E−34
WaOA vs. RSA	1.01E−24	2.29E−12	2.09E−26
WaOA vs. MPA	1.01E−24	5.98E−20	1.44E−34
WaOA vs. TSA	1.01E−24	0.044967	1.13E−05
WaOA vs. MVO	1.01E−24	3.17E−18	1.44E−34
WaOA vs. GWO	1.01E−24	1.17E−16	1.44E−34
WaOA vs. TLBO	1.01E−24	2.37E−13	1.44E−34
WaOA vs. GSA	1.01E−24	1.97E−21	3.22E−13
WaOA vs. PSO	1.01E−24	1.97E−21	5.35E−17
WaOA vs. GA	1.01E−24	1.49E−11	1.44E−34

### Sensitivity analysis

WaOA is a population-based optimizer that performs the optimization process in a repetitive-based calculation. Accordingly, the parameters $N$ (the number of members of the population) and $T$ (the total number of iterations of the algorithm) are expected to affect the WaOA optimization performance. Therefore, WaOA’s sensitivity analysis to parameters $T$ and $N$ is presented in this subsection.

**Figure 4:**
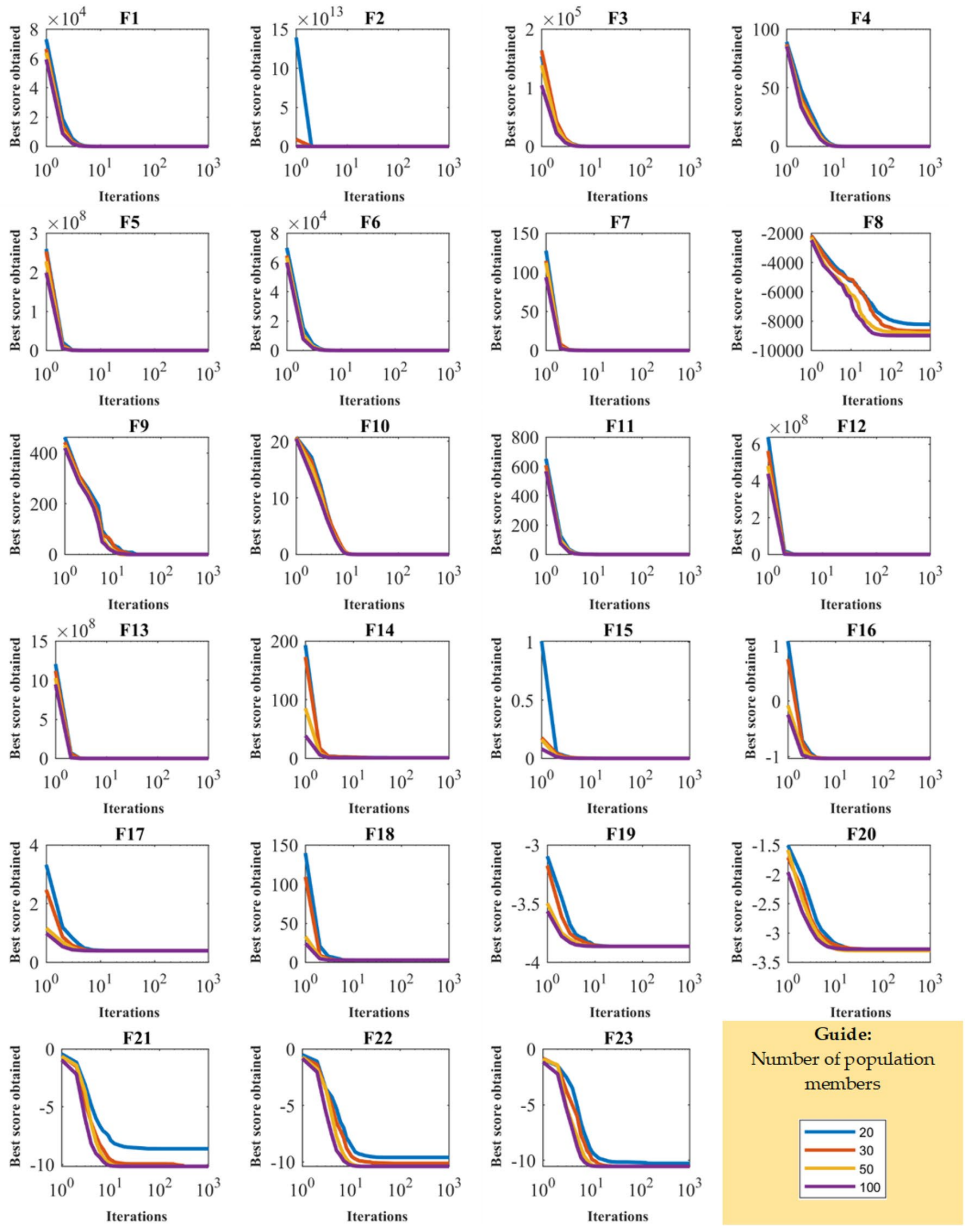
WaOA’s convergence curves in the study of sensitivity analysis to the parameter $N$.

For analyzing the sensitivity of WaOA to the parameter $N$, the proposed algorithm for different values of the parameter $N$ equal to 20, 30, 50, and 100 is used to optimize the functions of F1 to F23. Optimization results are given in Table 6, and WaOA’s convergence curves under this analysis are presented in Fig. 4. What is evident from the analysis of WaOA’s sensitivity to the parameter $N$ is that increasing the searcher agents improves WaOA’s search capability in scanning the search space, which enhances the performance of the proposed algorithm and reduces the values of the objective function.

**Table 6:** Results of WaOA sensitivity analysis to the parameter $N$.

Objective function	Number of population members			
	20	30	50	100
F_1_	0	0	0	0
F_2_	1.3E−287	1.9E−291	1.02E−294	3.6E−301
F_3_	0	0	0	0
F_4_	3.6E−268	2.5E−272	2.12E−277	1.1E−286
F_5_	0	0	0	0
F_6_	0	0	0.00E+00	0
F_7_	1.42E−05	1.65E−05	1.43E−05	6.27E−06
F_8_	− 8217.03	− 8671.19	− 8881.11	− 8955.43
F_9_	0	0	0.00E+00	0
F_10_	2.13E−15	3.38E−15	2.13E−15	2.13E−15
F_11_	0	0	0.00E+00	0
F_12_	1.57E−32	1.57E−32	1.57E−32	1.57E−32
F_13_	1.35E−32	1.35E−32	1.35E−32	1.35E−32
F_14_	0.998004	0.998004	0.998004	0.998004
F_15_	0.000359	0.000359	0.000307	0.000307
F_16_	− 1.03163	− 1.03163	− 1.03163	− 1.03163
F_17_	0.397887	0.397887	0.397887	0.397887
F_18_	3	3	3	3
F_19_	− 3.86278	− 3.86278	− 3.86278	− 3.86278
F_20_	− 3.29822	− 3.30444	− 3.322	− 3.27444
F_21_	− 8.6238	− 10.1532	− 10.1532	− 10.1532
F_22_	− 9.60565	− 10.1372	− 10.4029	− 10.4029
F_23_	− 10.266	− 10.5364	− 10.5364	− 10.5364

**Figure 5:**
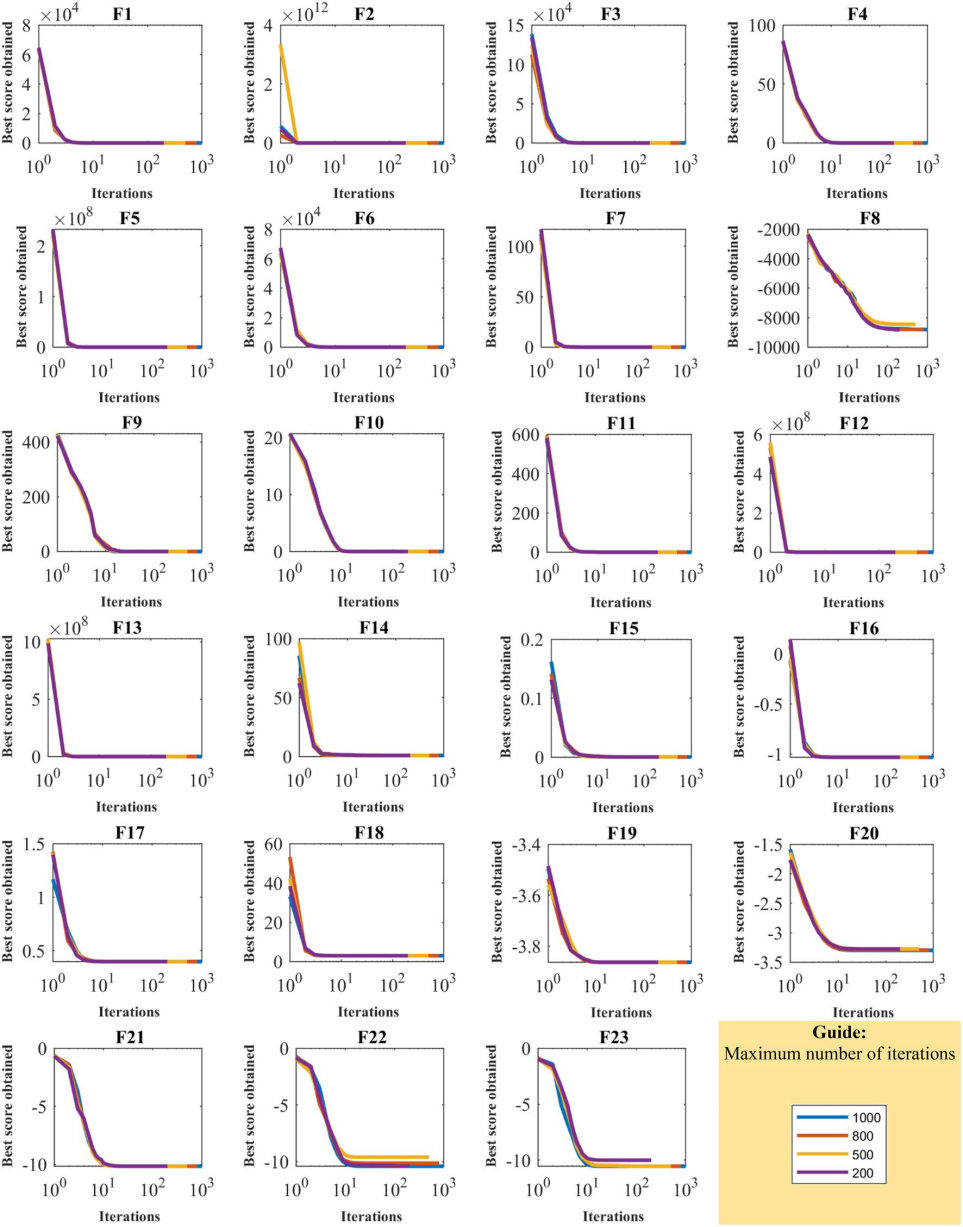
WaOA’s convergence curves in the study of sensitivity analysis to parameter $T$.

For analyzing the sensitivity of the proposed algorithm to the parameter $T$, WaOA for different values of the parameter $T$ equal to 200, 500, 800, and 1000 is used to optimize the functions of F1 to F23. Optimization results are in Table 7, and the WaOA’s convergence curves under this analysis are presented in Fig. 5. Based on the obtained results, it is found that increasing values of $T$ gives the algorithm more opportunity to converge to better solutions based on exploitation ability. Therefore, it can be seen that with increasing values of $T$, the optimization process has become more efficient, and as a result, the values of the objective function have decreased.

**Table 7:** Results of WaOA’s sensitivity analysis to the parameter $T$.

Objective function	Maximum number of iterations			
	200	500	800	1000
F_1_	1.4E−112	9.5E−287	0	0
F_2_	1.85E−57	6.7E−147	3.4E−236	1.02E−294
F_3_	6.04E−83	1.9E−217	0	0
F_4_	5.11E−54	2.7E−138	1.5E−222	2.12E−277
F_5_	0	0	0	0
F_6_	8.52E−05	0	0	0.00E+00
F_7_	7.89E−05	1.83E−05	2.1E−05	1.43E−05
F_8_	− 8822.99	− 8449.52	− 8792.24	− 8881.11
F_9_	0	0	0	0.00E+00
F_10_	2.84E−15	1.42E−15	2.31E−15	2.13E−15
F_11_	0	0	0	0.00E+00
F_12_	1.57E−32	1.57E−32	1.57E−32	1.57E−32
F_13_	1.35E−32	1.35E−32	1.35E−32	1.35E−32
F_14_	0.998004	0.998004	0.998004	0.998004
F_15_	0.000313	0.000307	0.000353	0.000307
F_16_	− 1.03163	− 1.03163	− 1.03163	− 1.03163
F_17_	0.397887	0.397887	0.397887	0.397887
F_18_	3	3	3	3
F_19_	− 3.86278	− 3.86278	− 3.86278	− 3.86278
F_20_	− 3.27444	− 3.27444	− 3.29227	− 3.322
F_21_	− 10.1532	− 10.1532	− 10.1532	− 10.1532
F_22_	− 10.4029	− 10.4029	− 10.4029	− 10.4029
F_23_	− 9.99562	− 10.5364	− 10.5364	− 10.5364

### Evaluation of the CEC 2015 test suite

The optimization results of the CEC 2015 test suite, including C15–F1 to C15–F15 using WaOA and competitor algorithms, are released in Table 8. The simulation results show that WaOA is the best optimizer for C15–F1 to C15–F8, C15–F10, C15–F13, and C15–F14 functions. In addition, in solving C15–F9 after MVO, in C15–F11 after WSO, C15–F12, and C15–F15 after GSA, the proposed WaOA is the second-best optimizer. Analysis of simulation results shows that WaOA provides better results in most functions of the CEC 2015 test suite, and in total, with the first rank of the best optimizer in handling the CEC 2015 test suite, has provided superior performance compared to competitor algorithms.

**Table 8:** Evaluation results of the CEC 2015 test suite functions.

	WaOA	WSO	RSA	MPA	TSA	MVO	GWO	TLBO	GSA	PSO	GA
C15–F1											
Avg	1.00E+02	4.78E+04	9.04E+07	7.08E+06	2.39E+07	1.28E+06	4.68E+06	4.26E+06	4.81E+06	6.10E+04	2.15E+07
Std	1.52E−07	3.50E+04	2.70E+07	3.39E+06	1.88E+07	4.76E+05	5.18E+06	2.29E+06	1.21E+06	5.59E+04	2.36E+07
Rank	1	2	11	8	10	4	6	5	7	3	9
C15–F2											
Avg	2.00E+02	1.01E+04	8.96E+09	4.16E+06	1.70E+09	1.57E+04	6.57E+06	9.88E+07	5.00E+03	3.06E+03	4.05E+06
Std	5.06E−06	2.89E+03	1.43E+09	2.32E+06	2.72E+09	8.00E+03	3.53E+06	2.77E+07	1.32E+03	4.98E+03	2.84E+06
Rank	1	4	11	7	10	5	8	9	3	2	6
C15–F3											
Avg	3.15E+02	3.20E+02	3.21E+02	3.20E+02	3.20E+02	3.20E+02	3.20E+02	3.20E+02	3.20E+02	3.20E+02	3.20E+02
Std	1.00E+01	5.70E−02	6.29E−02	8.88E−02	1.71E−01	1.23E−02	6.71E−02	5.39E−02	1.22E−05	3.40E−06	1.20E−01
Rank	1	10	11	5	8	4	9	7	2	3	6
C15–F4											
Avg	4.09E+02	4.16E+02	4.68E+02	4.47E+02	4.48E+02	4.25E+02	4.13E+02	4.36E+02	4.32E+02	4.18E+02	4.28E+02
Std	2.37E+00	5.77E−01	4.16E+00	1.83E+01	8.18E+00	8.54E+00	3.08E+00	1.44E+00	6.96E+00	8.18E+00	5.21E+00
Rank	1	3	11	9	10	5	2	8	7	4	6
C15–F5											
Avg	6.10E+02	1.11E+03	1.82E+03	1.71E+03	1.54E+03	1.19E+03	1.05E+03	1.59E+03	1.62E+03	1.38E+03	8.01E+02
Std	8.78E+01	5.73E+02	1.98E+02	2.44E+02	2.33E+02	1.17E+02	3.06E+02	1.45E+02	1.97E+02	4.01E+02	1.88E+02
Rank	1	4	11	10	7	5	3	8	9	6	2
C15–F6											
Avg	6.06E+02	8.59E+02	1.33E+06	4.45E+05	1.77E+04	5.66E+03	4.48E+04	2.34E+04	9.37E+04	3.97E+03	1.28E+05
Std	3.87E+00	1.65E+02	2.22E+06	3.72E+05	2.71E+04	3.44E+03	4.17E+04	2.80E+04	8.35E+04	4.50E+03	2.12E+05
Rank	1	2	11	10	5	4	7	6	8	3	9
C15–F7											
Avg	7.01E+02	7.02E+02	7.25E+02	7.05E+02	7.14E+02	7.02E+02	7.03E+02	7.04E+02	7.04E+02	7.03E+02	7.05E+02
Std	3.21E−01	1.04E+00	1.28E+01	1.63E+00	9.16E+00	7.26E−01	1.26E+00	8.04E−01	4.26E−01	1.45E+00	4.35E−01
Rank	1	2	11	9	10	3	5	6	7	4	8
C15–F8											
Avg	8.01E+02	8.66E+02	1.95E+05	1.17E+04	5.30E+05	6.72E+03	4.50E+03	3.27E+03	4.31E+05	8.05E+04	5.37E+05
Std	4.81E−01	4.56E+01	2.73E+05	8.05E+03	1.05E+06	7.65E+03	1.67E+03	7.52E+02	5.82E+05	1.52E+05	7.04E+05
Rank	1	2	8	6	10	5	4	3	9	7	11
C15–F9											
Avg	1.00E+03	1.00E+03	1.03E+03	1.00E+03	1.02E+03	1.00E+03	1.00E+03	1.00E+03	1.00E+03	1.00E+03	1.00E+03
Std	5.95E−02	5.37E−01	1.82E+00	2.33E−01	2.10E+01	9.06E−02	1.51E−01	9.66E−02	2.03E−01	5.05E−01	2.27E+00
Rank	2	8	11	6	10	1	3	5	4	7	9
C15–F10											
Avg	1.22E+03	1.29E+03	5.75E+04	1.47E+04	8.46E+03	2.45E+03	1.99E+03	4.48E+03	1.43E+05	2.28E+03	8.56E+03
Std	2.20E−01	6.41E+01	5.21E+04	1.91E+04	4.47E+03	1.58E+03	6.42E+02	9.19E+02	1.19E+05	6.70E+02	8.29E+03
Rank	1	2	10	9	7	5	3	6	11	4	8
C15–F11											
Avg	1.33E+03	1.26E+03	1.49E+03	1.53E+03	1.35E+03	1.43E+03	1.43E+03	1.33E+03	1.40E+03	1.45E+03	1.33E+03
Std	1.48E+02	1.65E+02	7.13E+01	1.42E+02	1.55E+02	6.58E+01	6.80E+01	1.40E+02	8.10E−01	1.09E+02	1.40E+02
Rank	2	1	10	11	5	7	8	3	6	9	4
C15–F12											
Avg	1.30E+03	1.31E+03	1.34E+03	1.31E+03	1.31E+03	1.30E+03	1.30E+03	1.31E+03	1.30E+03	1.30E+03	1.31E+03
Std	4.54E−01	4.00E+00	6.01E+00	5.81E+00	2.14E+01	4.61E−01	6.84E−01	1.28E+00	4.74E−01	1.11E+00	1.86E+00
Rank	2	7	11	8	10	4	3	6	1	5	9
C15–F13											
Avg	1.30E+03	1.30E+03	1.30E+03	1.30E+03	1.31E+03	1.30E+03	1.30E+03	1.30E+03	1.53E+03	1.30E+03	1.30E+03
Std	6.61E−05	8.56E−02	7.92E−02	9.65E−04	8.72E+00	1.89E−04	7.89E−05	9.94E−04	2.26E+02	2.44E−03	9.98E−01
Rank	1	8	7	4	10	3	2	5	11	6	9
C15–F14											
Avg	3.63E+03	3.68E+03	1.37E+04	8.39E+03	1.05E+04	5.37E+03	7.76E+03	7.13E+03	7.04E+03	4.72E+03	4.54E+03
Std	1.42E+03	1.41E+03	3.62E+03	1.54E+01	3.76E+03	4.16E+03	1.65E+03	3.33E+03	3.80E+03	6.28E+02	9.97E+02
Rank	1	2	11	9	10	5	8	7	6	4	3
C15–F15											
Avg	1.60E+03	1.60E+03	2.25E+03	1.60E+03	2.56E+03	1.60E+03	1.61E+03	1.61E+03	1.60E+03	1.61E+03	1.62E+03
Std	1.56E−06	5.86E+00	3.88E+02	1.14E+00	1.85E+03	8.24E-03	1.14E+01	1.98E+00	4.71E-10	1.19E+01	3.03E+00
Rank	2	4	10	5	11	3	8	7	1	6	9
Sum rank	19	61	155	116	133	63	79	91	92	73	108
Mean rank	1.2666667	4.0666667	10.333333	7.7333333	8.8666667	4.2	5.2666667	6.0666667	6.1333333	4.8666667	7.2
Total rank	1	2	11	9	10	3	5	6	7	4	8

### Evaluation of the CEC 2017 test suite

The employment results of WaOA and competitor algorithms on the CEC 2017 test suite including functions C17–F1 to C17–F30 are presented in Table 9. What can be seen from the analysis of the simulation results is that WaOA is the first best optimizer for C17–F1 to C17–F6, C17–F8 to C17–F30 functions. in solving C17–F7, proposed WaOA after GSA is the second-best optimizer. Comparison of simulation results shows that WaOA has provided better results in most functions of CEC 2017 test suite, and has provided superior performance in solving this test suite compared to competing algorithms.

**Table 9:** Evaluation results of the CEC 2017 test suite functions.

	WaOA	WSO	RSA	MPA	TSA	MVO	GWO	TLBO	GSA	PSO	GA
C17–F1											
Avg	100.00004	4005.6583	9.571E+09	4,504,091.7	5.069E+09	5898.8934	7,663,270.9	86,504,717	202.1255	2974.6435	13,576,411
Std	3.857E−05	5309.3214	2.312E+09	2,221,337.5	7.611E+09	1961.9458	15,264,926	25,190,665	115.01188	3068.4227	1,781,695.6
Rank	1	4	11	6	10	5	7	9	2	3	8
C17–F2											
Avg	200.00002	4228.1754	1.994E+10	37,313,852	1.455E+10	23,697.882	7,468,532.5	257,121,613	1255.1705	35,032,131	32,991,045
Std	1.18E−05	4865.4087	4.47E+09	56,709,504	1.041E+10	10,810.599	14,605,747	52,488,502	1160.2677	70,055,940	19,701,103
Rank	1	3	11	8	10	4	5	9	2	7	6
C17–F3											
Avg	300	300.66248	12,372.195	1538.0906	7983.9163	300.03174	4578.8062	970.53362	9455.1389	300	40,090.229
Std	1.573E−10	8.10E−01	3673.3763	823.4105	7228.3746	0.0177723	3242.8635	479.35917	3173.5597	5.684E−14	1.61E+04
Rank	2	4	10	6	8	3	7	5	9	1	11
C17–F4											
Avg	400	402.69156	1204.7711	479.68829	554.74363	403.83135	407.54618	430.18871	410.28556	402.6291	418.7015
Std	4.122E−07	2.9072717	624.83773	61.325713	90.662778	1.5737425	0.3059483	28.554211	8.7544971	3.7996053	3.0735855
Rank	1	3	11	9	10	4	5	8	6	2	7
C17–F5											
Avg	510.19832	511.44508	604.14973	567.45322	565.26475	522.7829	520.19777	536.33148	549.0015	538.55953	536.08736
Std	1.6992136	0.5709551	9.5964298	16.615571	8.2775098	15.06703	12.849548	5.835234	6.9587408	13.157491	5.0403291
Rank	1	2	11	10	9	4	3	6	8	7	5
C17–F6											
Avg	600.00031	601.71109	644.53432	634.05295	638.7989	603.06512	600.61836	612.47322	621.10746	610.33258	610.23111
Std	0.0001343	1.463828	9.7346987	12.368649	14.732547	3.339361	0.6472702	2.8673291	8.0147542	8.4990205	2.0708594
Rank	1	3	11	9	10	4	2	7	8	6	5
C17–F7											
Avg	717.64017	722.19608	800.7207	766.54081	822.42204	729.56658	732.19707	753.07534	716.49748	730.7263	742.2823
Std	2.1495234	8.303089	3.2023255	25.183691	16.346488	8.1279419	5.5558736	9.5461724	3.9191109	10.55981	1.6662753
Rank	2	3	10	9	11	4	6	8	1	5	7
C17–F8											
Avg	808.45715	811.95306	863.08934	835.50469	831.72026	827.1168	811.8188	831.86546	819.89915	823.38149	823.11839
Std	1.7233196	2.1495061	6.7267648	6.3444503	7.8313473	19.350475	4.3750491	6.9014827	3.1463198	6.9171262	5.2318773
Rank	1	3	11	10	8	7	2	9	4	6	5
C17–F9											
Avg	900	905.23523	1654.0566	1539.8706	1144.9518	900.0254	962.68206	932.13388	955.75337	926.84022	904.554
Std	1.683E−08	6.98E+00	2.84E+02	547.48016	86.402315	0.0434752	72.920311	29.975945	66.794413	42.552004	1.6048115
Rank	1	4	11	10	9	2	8	6	7	5	3
C17–F10											
Avg	1609.1526	1697.3584	2579.7024	2103.8934	2047.56	1766.1425	1628.3692	2487.3367	2738.2993	2016.4711	1680.1752
Std	63.444048	324.93072	306.17817	343.97201	167.25007	495.29733	36.706618	196.14215	414.01026	125.5258	195.94284
Rank	1	4	10	8	7	5	2	9	11	6	3
C17–F11											
Avg	1102.9976	1109.5838	5308.3512	1211.7691	1248.9475	1135.9375	1153.3651	1154.9136	1123.9687	1153.7295	1932.1708
Std	1.8340875	8.9302238	2509.2652	45.169228	134.61252	24.701296	56.570343	24.344365	8.2568248	10.621192	1460.7433
Rank	1	2	11	8	9	4	5	7	3	6	10
C17–F12											
Avg	1224.2329	6371.0574	323,042,178	4,940,009.8	4,337,558	6.74E+05	522,985.26	3,104,175.8	948,157.78	18,665.709	1,728,527.2
Std	19.82966	5237.7263	138,057,073	5,936,430.7	4,356,750.6	6.13E+05	523,173.23	2,091,217.6	1,265,852.4	12,647.309	2,601,729.6
Rank	1	2	11	10	9	5	4	8	6	3	7
C17–F13											
Avg	1304.3998	1436.2569	3,433,392.8	13,523.963	14,864.261	1.50E+04	7408.9458	15,870.564	12,818.438	8543.6896	17,948.699
Std	1.8121278	85.771613	3,712,009.2	11,827.768	6748.8206	1.26E+04	8552.8328	1481.2216	2473.5339	5115.9967	12,398.879
Rank	1	2	11	6	7	8	3	9	5	4	10
C17–F14											
Avg	1402.7375	1447.3093	16,184.889	2824.7697	3410.3822	1442.5054	3315.0362	1552.051	6218.272	2979.7936	5062.1231
Std	0.9531651	17.56891	15,042.221	1783.7714	2195.5301	16.313511	2077.2433	28.205894	929.37787	1240.587	6330.4778
Rank	1	3	11	5	8	2	7	4	10	6	9
C17–F15											
Avg	1500.457	1520.2257	12,335.319	8356.3696	6352.9142	1841.005	6220.8329	1680.1684	17,291.842	4169.7606	3902.2726
Std	0.1910035	14.358268	8013.0628	9589.7211	3732.9251	633.67594	1192.3378	37.759578	6993.8156	2919.2862	2148.9119
Rank	1	2	10	9	8	4	7	3	11	6	5
C17–F16											
Avg	1601.3414	1666.8615	2044.7313	1977.7889	1879.6084	1732.9873	1733.6205	1715.3545	2123.5173	1881.8063	1821.5272
Std	0.2819816	73.125749	77.078212	260.53269	116.61807	160.42116	104.31509	111.73755	99.236078	168.24657	58.360282
Rank	1	2	10	9	7	4	5	3	11	8	6
C17–F17											
Avg	1722.8854	1744.8219	1840.2169	1772.1101	1851.4808	1756.9088	1772.6013	1777.1329	1835.1183	1784.4765	1754.133
Std	1.2581607	13.521179	38.112673	28.70145	104.05434	52.167653	50.02034	27.487099	101.85986	66.170275	5.5600511
Rank	1	2	10	5	11	4	6	7	9	8	3
C17–F18											
Avg	1801.4365	1829.7721	99,279,162	27,952.74	35,640.588	13,759.79	28,891.102	47,436.515	5694.8466	12,575.652	12,737.378
Std	1.3307674	17.083301	138,699,482	17,630.376	21,701.121	10,600.924	20,917.048	20,041.434	4745.3429	16,839.651	8104.9154
Rank	1	2	11	7	9	6	8	10	3	4	5
C17–F19											
Avg	1900.7242	1903.9714	527,934.93	27,988.531	77,738.078	2193.5018	8215.5575	2025.1666	36,470.027	61,702.038	4801.2686
Std	0.193972	3.2499933	636,547.59	20,663.325	134,371.74	433.48789	8056.2529	39.983743	12,447.72	101,179.04	5033.0595
Rank	1	2	11	7	10	4	6	3	8	9	5
C17–F20											
Avg	2010.7371	2064.4114	2301.9518	2189.5507	2160.5974	2151.2179	2047.7079	2085.9689	2365.5044	2148.9694	2056.9687
Std	10.208906	57.733768	25.000475	88.30438	70.431939	26.93479	16.640885	18.109296	131.50071	51.212021	4.5614343
Rank	1	4	10	9	8	7	2	5	11	6	3
C17–F21											
Avg	2200	2290.8044	2346.8044	2352.0055	2317.1897	2319.8346	2314.2228	2272.5201	2351.4715	2336.4486	2227.6461
Std	6.913E−06	44.47952	72.243642	25.285867	69.593129	9.1135999	3.2521393	76.685514	30.582882	2.2640433	10.653304
Rank	1	4	9	11	6	7	5	3	10	8	2
C17–F22											
Avg	2250.496	2312.7444	2996.635	2314.3395	2533.9327	2303.6528	2306.5265	2333.2749	2300.0861	2314.0531	2316.0897
Std	58.307723	6.0920769	180.07862	17.751045	100.92037	1.2728103	2.6166121	10.956332	0.1722197	24.064845	3.4298382
Rank	1	5	11	7	10	3	4	9	2	6	8
C17–F23											
Avg	2610.776	2635.008	2689.6255	2650.6824	2754.766	2618.9319	2621.7636	2632.4649	2756.311	2657.0042	2661.2858
Std	2.9977776	13.912924	11.692104	19.466531	45.976842	6.4893472	15.056006	4.3053546	71.277096	41.148475	11.807443
Rank	1	5	9	6	10	2	3	4	11	7	8
C17–F24											
Avg	2500.0003	2682.498	2870.0973	2771.212	2834.0566	2754.0268	2741.1157	2763.4569	2652.4055	2632.2162	2654.7784
Std	0.000142	121.66169	31.220112	12.686776	39.395546	7.1079038	10.523085	6.7503475	176.04108	152.7966	149.58103
Rank	1	5	11	9	10	7	6	8	3	2	4
C17–F25											
Avg	2897.7436	2935.6309	3343.3736	2951.7858	3087.8795	2921.0788	2928.416	2941.3726	2932.6657	2909.618	2954.021
Std	0.0014007	25.405188	59.618626	33.467254	373.67949	26.250929	14.749164	17.883979	22.027622	23.135637	3.1906326
Rank	1	6	11	8	10	3	4	7	5	2	9
C17–F26											
Avg	2825.0016	3025.6385	4345.855	3648.1255	3431.5898	3170.0765	2886.8783	2976.3185	3624.8912	3420.5334	3069.0681
Std	49.998967	104.41524	106.17494	585.55715	354.13465	539.96121	92.382389	29.400728	708.34009	727.58805	35.394045
Rank	1	4	11	10	8	6	2	3	9	7	5
C17–F27											
Avg	3089.2093	3120.0204	3158.1751	3128.4995	3161.7517	3108.5107	3112.2837	3108.2979	3252.8976	3136.9496	3141.3768
Std	0.2503956	22.336942	30.215021	34.632185	61.440793	28.654321	41.837057	23.994805	6.1472642	38.942622	32.540974
Rank	1	5	9	6	10	3	4	2	11	7	8
C17–F28											
Avg	3100.0001	3173.5788	3740.2471	3535.0128	3627.3232	3264.0278	3423.2143	3437.5636	3478.1634	3279.8176	3359.2612
Std	3.817E−05	140.20046	197.0247	154.22247	80.831235	156.81375	12.61691	213.69409	12.226383	147.67403	1.56E+02
Rank	1	2	11	9	10	3	6	7	8	4	5
C17–F29											
Avg	3143.6425	3188.9391	3437.8261	3361.7066	3309.3923	3226.1848	3191.8576	3180.659	3345.3659	3248.8239	3220.4056
Std	13.341168	12.648429	200.8649	94.237596	158.43895	35.134243	20.713203	36.903709	137.98739	62.700717	30.026932
Rank	1	3	11	10	8	6	4	2	9	7	5
C17–F30											
Avg	3396.3409	3932.8711	9,969,841	1,378,673.9	4,145,442	38,155.39	868,418.89	905,676.07	1,427,094.7	10,054.76	1,317,197.3
Std	0.846773	301.84652	7,022,481.1	1,278,684	3,562,683.3	2.66E+04	812,465.74	875,023.24	477,453.09	4035.7159	1,114,518.6
Rank	1	2	11	8	10	4	5	6	9	3	7
Sum rank	32	97	317	244	270	134	143	186	212	161	184
Mean rank	1.0666667	3.2333333	10.566667	8.1333333	9	4.4666667	4.7666667	6.2	7.0666667	5.3666667	6.1333333
Total rank	1	2	11	9	10	3	4	7	8	5	6

### Informed consent

Informed consent was not required as no human or animals were involved.

### Ethical approval

This article does not contain any studies with human participants or animals performed by any of the authors.

## WaOA’s application to real-world problems

Metaheuristic algorithms are one of the most widely used techniques in dealing with real-world applications. This section tests WaOA performance in optimizing four engineering design challenges and twenty-two constrained optimization problems from the CEC 2011 test suite. It should be noted that to model the constraints of optimization problems, the penalty function has been used. Thus, if a solution does not meet any of the constraints of the problem, a penalty coefficient is added to the value of its objective function corresponding to each non-compliance of the constraint, and as a result, it is known as an inappropriate solution.

### tension/compression spring design optimization problem

**Figure 6:**
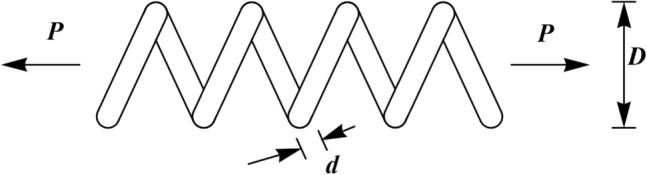
Schematic view of the tension/compression spring problem.

Tension/compression spring design is a challenge in real-world applications with the aim of minimizing the weight of tension/compression spring. A schematic of this design is shown in Fig. 6^59^. The tension/compression spring problem formulation is as follows:

Consider $$X=\left[{x}_{1}, {x}_{2}, {x}_{3} \right]=\left[d, D, P\right].$$

Minimize $$f \left(X\right)=\left({x}_{3}+2\right){x}_{2}{x}_{1}^{2}.$$

Subject to:

$${g}_{1} \left(X\right)= 1-\frac{{x}_{2}^{3}{x}_{3}}{71785{x}_{1}^{4}} \le 0, \, \, {g}_{2} \left(X\right)=\frac{4{x}_{2}^{2}-{x}_{1}{x}_{2}}{12566({x}_{2}{x}_{1}^{3})}+\frac{1}{5108{x}_{1}^{2}}-1\le 0,$$

$${g}_{3} \left(X\right)= 1-\frac{140.45{x}_{1}}{{x}_{2}^{2}{x}_{3}}\le 0, \, \, {g}_{4} \left(X\right)=\frac{{x}_{1}+{x}_{2}}{1.5}-1 \le 0.$$

With.

$$0.05\le {x}_{1}\le 2, {0.25\le x}_{2}\le 1.3\mathrm{ and }2\le {x}_{3}\le 15$$.

**Figure 7:**
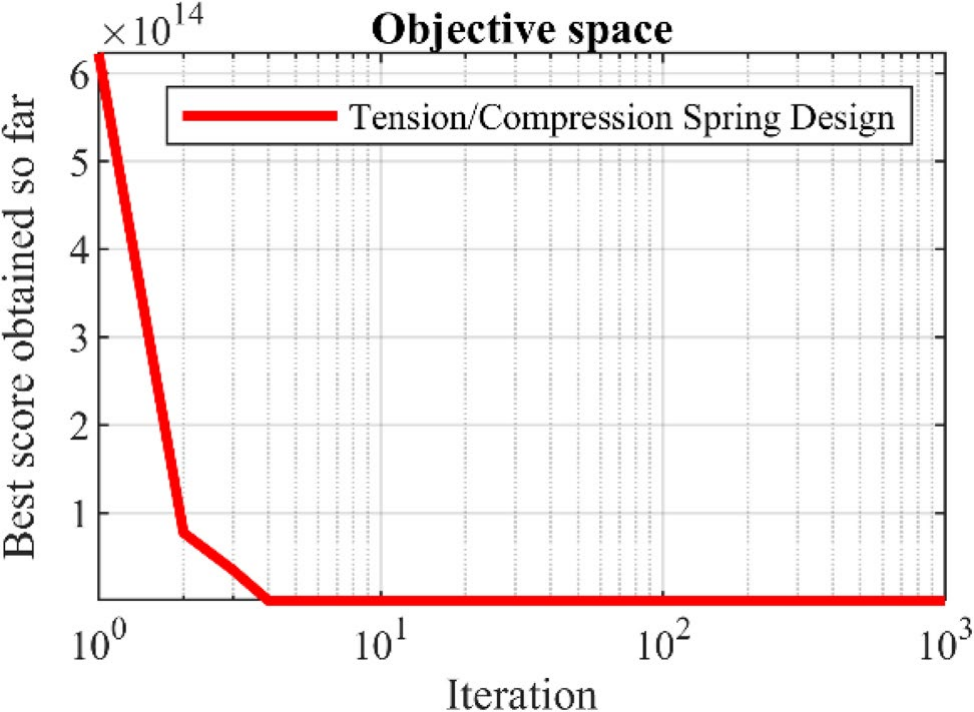
Convergence analysis of the WaOA for the tension/compression spring design optimization problem.

The results of using WaOA and competing algorithms in optimizing the Tension/compression spring design variables are presented in Table 10. The simulation results show that WaOA has provided the optimal solution to this problem with the values of the variables equal to (0.0519693, 0.363467, 10.9084) and the corresponding objective function value equal to 0.012672. The statistical results obtained from the performance of WaOA and competitor algorithms are reported in Table 11, which shows the superiority of WaOA in providing better values for statistical indicators. The WaOA convergence curve while achieving the solution for tension/compression spring is shown in Fig. 7.

**Table 10:** Comparison results for the tension/compression spring design problem.

Algorithm	Optimum variables			Optimum cost
	*d*	*D*	*P*	
WaOA	0.0519693	0.363467	10.9084	0.012672
WSO	0.057641	0.583026	14.00465	0.012722
RSA	0.051734	0.360336	11.54961	0.01317
MPA	0.050657	0.340484	11.98053	0.012782
TSA	0.049701	0.338294	11.95873	0.012786
MVO	0.049525	0.307463	14.85743	0.013305
GWO	0.049525	0.312953	14.09102	0.012926
TLBO	0.050297	0.331597	12.60176	0.012818
GSA	0.049525	0.314295	14.09343	0.012983
PSO	0.049624	0.307163	13.86693	0.013147
GA	0.049772	0.313344	15.09475	0.012885

**Table 11:** Statistical results for the tension/compression spring design problem.

Algorithm	Best	Mean	Worst	Std. dev	Median
WaOA	0.012672	0.012701	0.012706	0.001106	0.012700
WSO	0.012722	0.012754	0.012766	0.007391	0.012744
RSA	0.01317	0.013848	0.015774	0.006119	0.013727
MPA	0.012782	0.012799	0.01283	0.00567	0.012802
TSA	0.012786	0.012812	0.012836	0.004191	0.012815
MVO	0.013305	0.014951	0.018023	0.002293	0.013312
GWO	0.012926	0.014594	0.018	0.001636	0.014147
TLBO	0.012818	0.012956	0.013116	0.007828	0.012961
GSA	0.012983	0.01356	0.01434	0.000289	0.013488
PSO	0.013147	0.014162	0.016398	0.002092	0.013119
GA	0.012885	0.013188	0.015352	0.000378	0.013069

### Welded beam design

**Figure 8:**
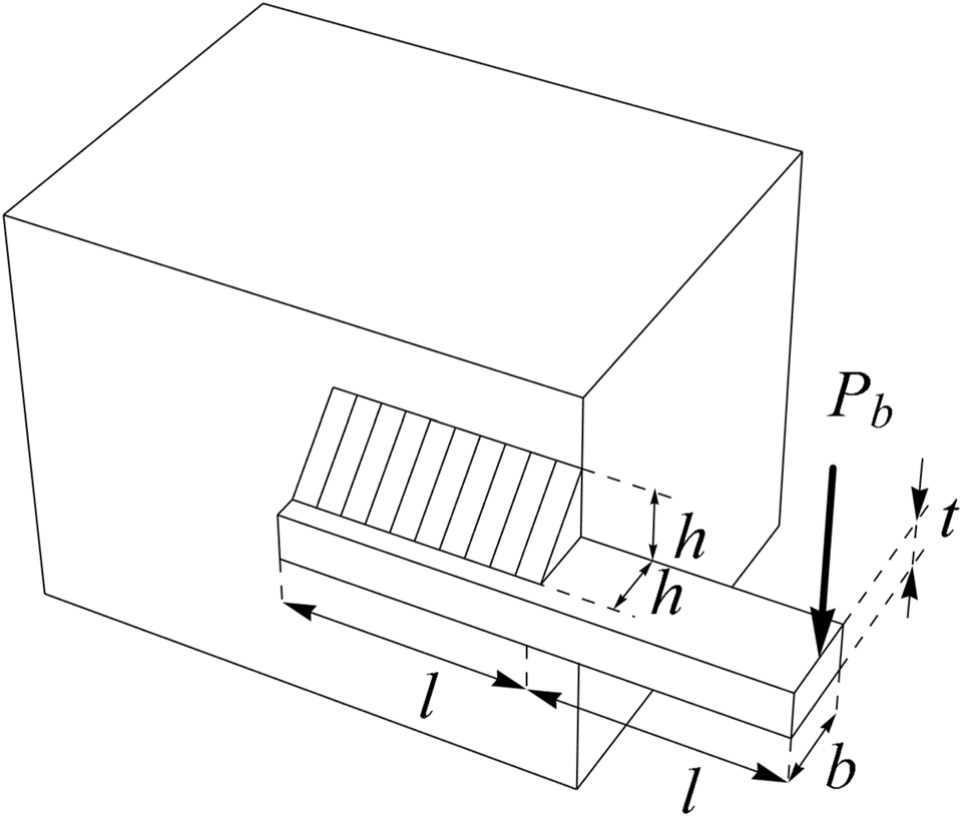
Schematic view of the welded beam design problem.

Welded beam design is a real global challenge in engineering sciences whose main goal in design is to reduce the fabrication cost of the welded beam. A schematic of this design is shown in Fig. 8^60^. The formulation of welded beam design problem is as follows:

Consider $$X=\left[{x}_{1}, {x}_{2}, {x}_{3}, {x}_{4}\right]=\left[h, l, t, b\right]$$.

Minimize $$f (X)=1.10471{x}_{1}^{2}{x}_{2}+0.04811{x}_{3}{x}_{4} (14.0+{x}_{2})$$.

Subject to:

$${g}_{1} \left(X\right)= \tau \left(X\right)-13600 \le 0, \,\, {g}_{2} \left(X\right)= \sigma \left(X\right)-30000 \le 0,$$

$${g}_{3} \left(X\right)= {x}_{1}-{x}_{4}\le 0, \,\, {g}_{4} (X) = 0.10471{x}_{1}^{2}+0.04811{x}_{3}{x}_{4} (14+{x}_{2})-5.0 \le 0,$$

$${g}_{5}\left(X\right)= 0.125 - {x}_{1}\le 0, \,\, {g}_{6}(X) = \delta (X) - 0.25 \le 0, \,\, {g}_{7} (X) = 6000 - {p}_{c} (X) \le 0.$$

*where*

$$\tau \left(X\right)=\sqrt{{\left({\tau }^{^{\prime}} \right)}^{2}+\left(2\tau {\tau }^{^{\prime}} \right)\frac{{x}_{2}}{2R}+{\left(\tau "\right)}^{2}}, \, \, { \tau }^{^{\prime}}=\frac{6000}{\sqrt{2}{x}_{1}{x}_{2}}, \,\, \tau "=\frac{MR}{J}$$

$$M=6000\left(14+\frac{{x}_{2}}{2}\right), \,\, R=\sqrt{\frac{{x}_{2}^{2}}{4}+{\left(\frac{{x}_{1}+{x}_{3}}{2}\right)}^{2}}, \,\, J=2\sqrt{2}{x}_{1}{x}_{2}\left(\frac{{x}_{2}^{2}}{12}+{\left(\frac{{x}_{1}+{x}_{3}}{2}\right)}^{2}\right)$$

$$\sigma \left(X\right)=\frac{\mathrm{504,000}}{{x}_{4}{x}_{3}^{2}}, \,\, \delta \left(x\right)=\frac{\mathrm{65,856,000}}{\left(30\cdot 1{0}^{6}\right){x}_{4}{x}_{3}^{3}}$$

$${p}_{c} \left(X\right)=\frac{4.013\left(30\cdot 1{0}^{6}\right){x}_{3}{x}_{4}^{3}}{3\cdot 196}\left(1-\frac{{x}_{3}}{28}\sqrt{\frac{30\cdot 1{0}^{6}}{4(12\cdot 1{0}^{6})}}\right).$$

With

$$0.1\le {x}_{1}, {x}_{4}\le 2 \, \mathrm{and} \, 0.1\le {x}_{2}, {x}_{3}\le 10.$$

**Figure 9:**
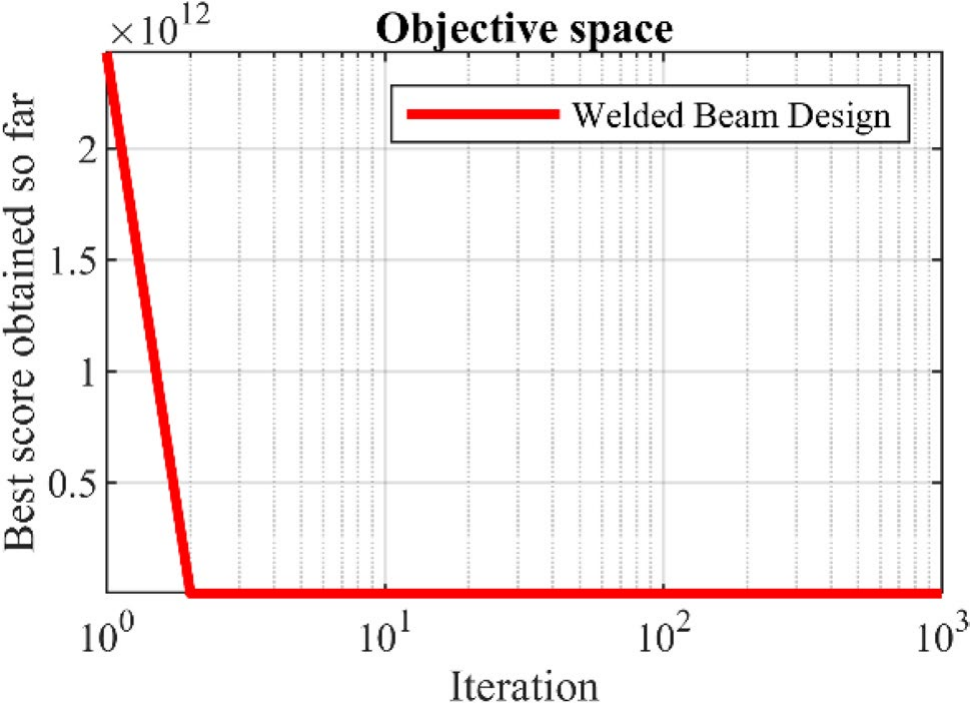
Convergence analysis of the WaOA for the welded beam design optimization problem.

WaOA and competing algorithms are implemented on the welded beam design problem, and the results are presented in Table 12. Based on these results, WaOA has provided the optimal solution to this problem with the values of the variables equal to (0.20573, 3.470489, 9.036624, 0.20573) and the corresponding objective function value equal to 1.724901. Statistical results from the performance of WaOA and competitor algorithms are reported in Table 13. This table shows that WaOA performs better in terms of statistical indicators. The convergence curve from the WaOA implementation on the welded beam design is shown in Fig. 9.

**Table 12:** Comparison results for the welded beam design problem.

Algorithm	Optimum variables				Optimum cost
	*h*	*l*	*t*	*b*	
WaOA	0.20573	3.470489	9.036624	0.20573	1.724901
WSO	0.205721	3.470747	9.037504	0.205721	1.725082
RSA	0.218482	3.510591	8.873427	0.224932	1.866307
MPA	0.205604	3.475541	9.037606	0.205852	1.728002
TSA	0.205719	3.476098	9.03877	0.20627	1.729338
MVO	0.19745	3.315724	10.0000	0.201435	1.822865
GWO	0.205652	3.472796	9.042739	0.20575	1.727813
TLBO	0.204736	3.536998	9.006091	0.210067	1.761559
GSA	0.147127	5.491842	10.0000	0.217769	2.175806
PSO	0.164204	4.033348	10.0000	0.223692	1.876513
GA	0.206528	3.636599	10.0000	0.20329	1.838741

**Table 13:** Statistical results for the welded beam design problem.

Algorithm	Best	Mean	Worst	Std. dev	Median
WaOA	1.724901	1.7270245	1.731028	0.005142	1.724508
WSO	1.725082	1.727023	1.725022	0.007133	1.726027
RSA	1.866307	1.892247	2.01658	0.007961	1.883728
MPA	1.728002	1.729207	1.729443	0.000287	1.729166
TSA	1.729338	1.730509	1.730945	0.001159	1.730468
MVO	1.822865	2.234675	3.054198	0.325161	2.249057
GWO	1.727813	1.733066	1.74506	0.004876	1.730801
TLBO	1.761559	1.821214	1.877075	0.027597	1.823691
GSA	2.175806	2.549219	3.009536	0.25636	2.499998
PSO	1.876513	2.123388	2.324666	0.034888	2.101153
GA	1.838741	1.366196	2.039231	0.139758	1.939537

### Speed reducer design

**Figure 10:**
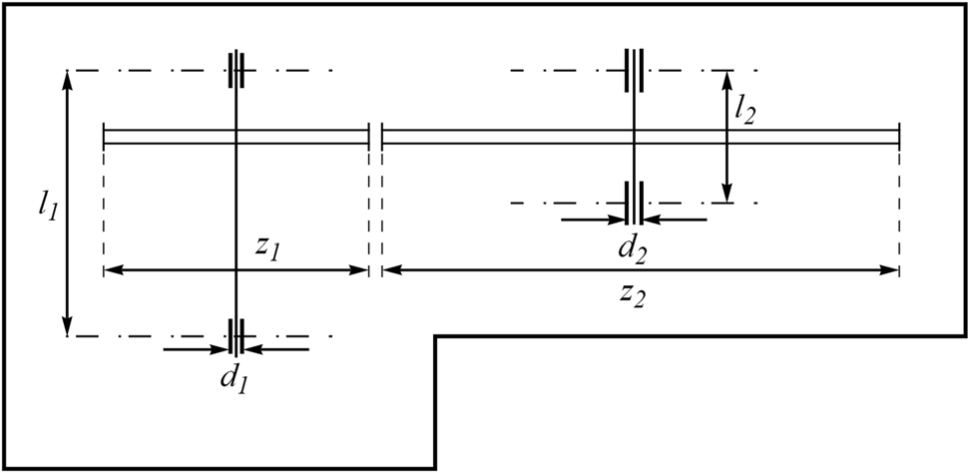
Schematic view of the speed reducer design problem.

Speed reducer design is a real-world engineering optimization challenge aimed at minimizing the weight of the speed reducer. A schematic of this design is shown in Fig. 10^61,62^. The speed reducer design problem is formulated as follows:

Consider $$X=\left[{x}_{1,} {x}_{2}, {x}_{3}, {x}_{4}, {x}_{5}{ ,x}_{6} ,{x}_{7}\right]=\left[b, m, p, {l}_{1}, {l}_{2}, {d}_{1}, {d}_{2}\right]$$.

Minimize $$f \left(X\right)=0.7854{x}_{1}{x}_{2}^{2}\left(3.3333{x}_{3}^{2}+14.9334{x}_{3}-43.0934\right)-1.508{x}_{1}\left({x}_{6}^{2}+{x}_{7}^{2}\right)+7.4777\left({x}_{6}^{3}+{x}_{7}^{3}\right)+0.7854({x}_{4}{x}_{6}^{2}+{x}_{5}{x}_{7}^{2})$$.

Subject to:

$${g}_{1} \left(X\right)=\frac{27}{{x}_{1}{x}_{2}^{2}{x}_{3}}-1 \le 0, \,\, {g}_{2} \left(X\right)=\frac{397.5}{{x}_{1}{x}_{2}^{2}{x}_{3}}-1\le 0,\,\, {g}_{3} \left(X\right)=\frac{1.93{x}_{4}^{3}}{{x}_{2}{x}_{3}{x}_{6}^{4}}-1\le 0,$$

$${g}_{4} \left(X\right)=\frac{1.93{x}_{5}^{3}}{{x}_{2}{x}_{3}{x}_{7}^{4}}-1 \le 0, \,\, {g}_{5}\left(X\right)=\frac{1}{110{x}_{6}^{3}}\sqrt{{\left(\frac{745{x}_{4}}{{x}_{2}{x}_{3}}\right)}^{2}+16.9\cdot {10}^{6}}-1\le 0,$$

$${g}_{6}(X) = \frac{1}{85{x}_{7}^{3}}\sqrt{{\left(\frac{745{x}_{5}}{{x}_{2}{x}_{3}}\right)}^{2}+157.5\cdot {10}^{6}}-1 \le 0,$$

$${g}_{7} \left(X\right)=\frac{{x}_{2}{x}_{3}}{40}-1 \le 0, \,\, {g}_{8} \left(X\right)=\frac{{5x}_{2}}{{x}_{1}}-1 \le 0, \,\, {g}_{9} \left(X\right)=\frac{{x}_{1}}{12{x}_{2}}-1 \le 0,$$

$${g}_{10} \left(X\right)=\frac{{1.5x}_{6}+1.9}{{x}_{4}}-1 \le 0, \,\, {g}_{11} \left(X\right)=\frac{{1.1x}_{7}+1.9}{{x}_{5}}-1 \le 0.$$

With

$$2.6\le {x}_{1}\le 3.6, 0.7\le {x}_{2}\le 0.8, 17\le {x}_{3}\le 28, 7.3\le {x}_{4}\le 8.3, 7.8\le {x}_{5}\le 8.3, 2.9\le {x}_{6}\le 3.9, \,\, \mathrm{and } \,\, 5\le {x}_{7}\le 5.5 .$$

**Figure 11:**
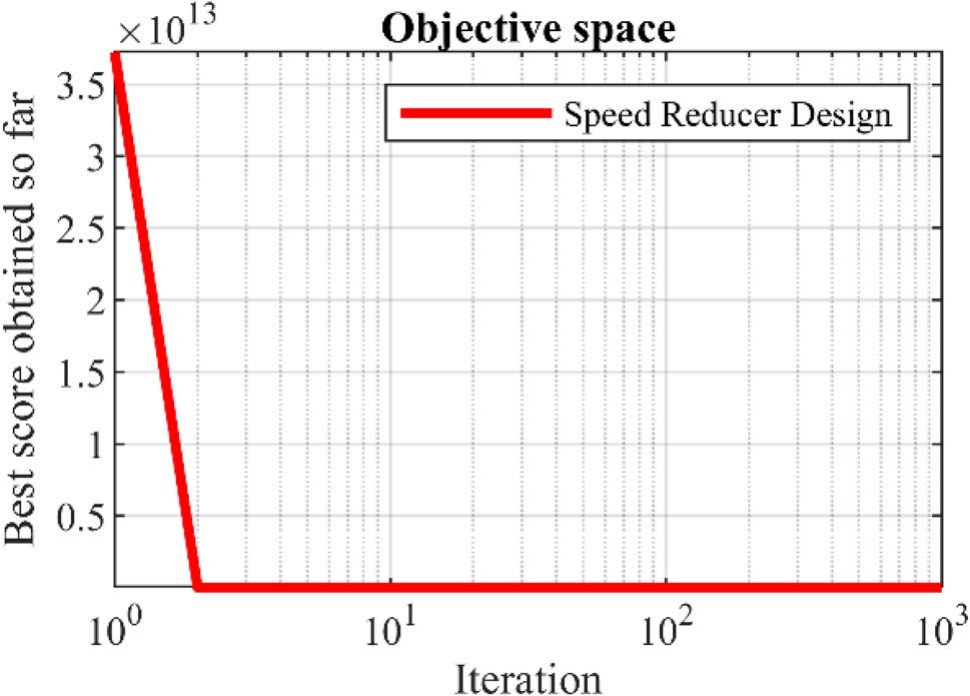
Convergence analysis of the WaOA for the speed reducer design optimization problem.

The results obtained by employing WaOA and competitor algorithms in speed reducer design optimization are reported in Table 14. The results show that WaOA has provided the optimal solution to this problem with the values of the variables equal to (3.5, 0.7, 17, 7.3, 7.8, 3.35021, 5.28668) and the corresponding objective function value equal to 2996.3482. The statistical results obtained from WaOA and the algorithms compared in Table 15 are released, which indicates the superiority of the proposed WaOA. The WaOA convergence curve while getting the solution to the speed reducer design problem is shown in Fig. 11.

**Table 14:** Comparison results for the speed reducer design problem.

Algorithm	Optimum variables							Optimum cost
	*b*	*m*	*p*	*l*_1_	*l*_2_	*d*_1_	*d*_2_	
WaOA	3.50000	0.700007	17	7.3	7.8	3.350209	5.286683	2996.3482
WSO	3.504191	0.70028	17.0068	7.311043	7.750249	3.35201	5.288865	2997.714
RSA	3.510772	0.70028	17.0068	7.399095	7.803283	3.361271	5.291898	3006.339
MPA	3.498013	0.698879	16.97279	7.288825	7.787514	3.347813	5.283283	2997.045
TSA	3.503107	0.698879	16.97279	7.36976	7.80327	3.354383	5.281313	2999.781
MVO	3.496443	0.698879	16.97279	8.287294	7.787569	3.348954	5.281261	3004.253
GWO	3.504918	0.698879	16.97279	7.398892	7.803577	3.354609	5.281322	3001.42
TLBO	3.50517	0.698879	16.97279	7.288316	7.787514	3.45745	5.283757	3029.041
GSA	3.596322	0.698879	16.97279	8.287294	7.787514	3.366182	5.283768	3049.589
PSO	3.506667	0.698879	16.97279	8.337218	7.787514	3.358732	5.282268	3066.02
GA	3.516528	0.698879	16.97279	8.357187	7.787514	3.363496	5.283262	3027.481

**Table 15:** Statistical results for the speed reducer design problem.

Algorithm	Best	Mean	Worst	Std. dev	Median
WaOA	2996.3482	2999.4961	3000.972	1.2463198	2998.6108
WSO	2997.714	3003.365	3008.597	5.221708	3001.932
RSA	3006.339	3013.236	3028.83	10.37327	3011.845
MPA	2997.045	2999.033	3003.281	1.931539	2999.979
TSA	2999.781	3005.237	3008.143	5.836758	3003.911
MVO	3004.253	3104.623	3210.524	79.62197	3104.623
GWO	3001.42	3028.228	3060.338	13.01596	3026.419
TLBO	3029.041	3065.296	3104.15	18.07054	3064.988
GSA	3049.589	3169.692	3363.192	92.55386	3156.113
PSO	3066.02	3185.877	3312.529	17.11513	3197.539
GA	3027.481	3294.662	3618.732	57.01195	3287.991

### Pressure vessel design

**Figure 12:**
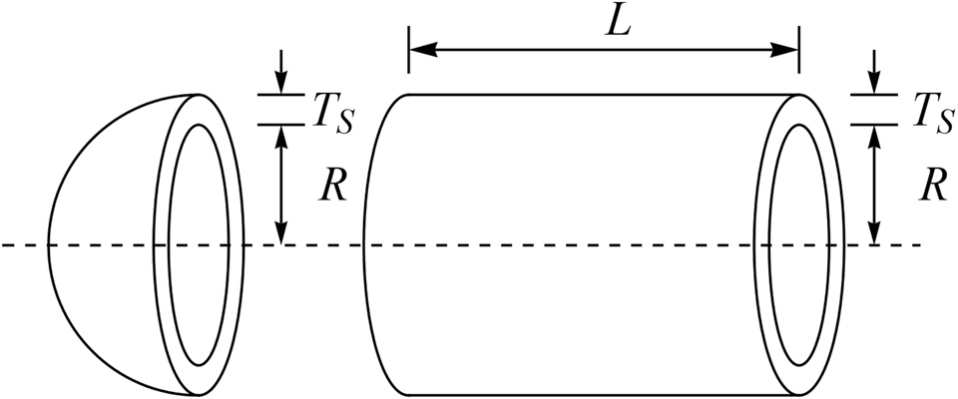
Schematic view of the pressure vessel design problem.

Pressure vessel design is a real-world optimization challenge that aims to reduce design costs. A schematic of this design is shown in Fig. 12^63^. The formulation of pressure vessel design problem is as follows:

Consider $$X=\left[{x}_{1}, {x}_{2}, {x}_{3}, {x}_{4}\right]=\left[{T}_{s}, {T}_{h}, R, L\right]$$.

Minimize $$f \left(X\right)=0.6224{x}_{1}{x}_{3}{x}_{4}+1.778{x}_{2}{x}_{3}^{2}+3.1661{x}_{1}^{2}{x}_{4}+19.84{x}_{1}^{2}{x}_{3}.$$

Subject to:

$${g}_{1} \left(X\right)= -{x}_{1}+0.0193{x}_{3} \le 0,\,\, {g}_{2} \left(X\right)=-{x}_{2}+0.00954{x}_{3}\le 0,$$

$${g}_{3} \left(X\right)=-\pi {x}_{3}^{2}{x}_{4}-\frac{4}{3}\pi {x}_{3}^{3}+1296000\le 0, \,\, {g}_{4} \left(X\right)={x}_{4}-240 \le 0.$$

With

$$0\le {x}_{1},{x}_{2}\le 100, \,\, {\mathrm{and} \,\, 10\le x}_{3},{x}_{4}\le 200.$$

**Figure 13:**
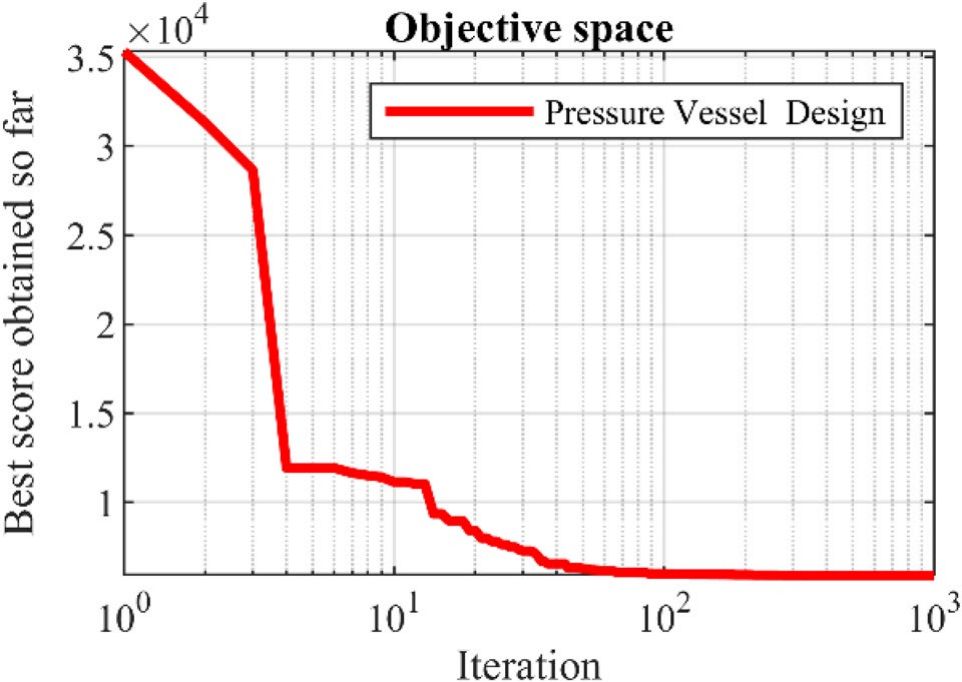
Convergence analysis of the WaOA for the pressure vessel design optimization problem.

WaOA and competitor algorithms are used in optimizing pressure vessel design. The results obtained for the design variables of this topic are released in Table 16. Based on this table, WaOA provides the optimal values of the design variables equal to (0.7782641, 0.3847753, 40.32163, 199.8713), which leads to the value equal to 5883.9604 for the objective function. The statistical indicators results obtained of performances of WaOA and competitor algorithms are presented in Table 17. Statistical results indicate that WaOA has effectively optimized the pressure vessel design challenge by providing more favorable values for statistical indicators. The WaOA convergence curve in achieving the optimal solution is shown in Fig. 13.

**Table 16:** Comparison results for the pressure vessel design problem.

Algorithm	Optimum variables				Optimum cost
	*T*_*s*_	*T*_*h*_	*R*	*L*	
WaOA	0.778264	0.384775	40.32163	199.8713	5883.9604
WSO	0.836709	0.417284	43.20765	160.9094	6010.62
RSA	0.810993	0.443429	42.0335	175.915	6088.866
MPA	0.795294	0.393338	41.20008	200	5909.092
TSA	0.796137	0.393104	41.21311	200	5912.899
MVO	0.826206	0.444881	42.80841	179.6187	5914.925
GWO	0.864286	0.427753	44.77817	159.8146	6035.531
TLBO	0.835526	0.427107	42.66592	187.6027	6161.892
GSA	1.109637	0.970461	50.4285	173.2081	11,596.44
PSO	0.768879	0.408312	41.34057	202.3494	5913.862
GA	1.123661	0.926481	45.43235	183.6029	6576.192

**Table 17:** Statistical results for the pressure vessel design problem.

Algorithm	Best	Mean	Worst	Std. dev	Median
WaOA	5884.8824	5887.201	5894.172	21.041638	5886.401
WSO	6010.62	6017.883	6021.73	31.07972	6015.981
RSA	6088.866	6096.722	6107.989	38.11009	6094.545
MPA	5909.092	5913.984	5918.883	29.06042	5912.763
TSA	5912.899	5918.082	5921.196	13.97272	5917.204
MVO	5914.925	6092.358	7427.921	66.91891	6445.037
GWO	6035.531	6506.504	7283.603	328.4812	6426.319
TLBO	6161.892	6355.281	6541.711	127.1797	6346.8
GSA	11,596.44	6871.379	7191.564	5816.728	6868.456
PSO	5913.862	6292.242	7037.332	498.3645	6140.245
GA	6576.192	6673.937	8041.527	660.4871	7620.206

### Evaluation of twenty-two real-world optimization problems from the CEC 2011 test suite

**Figure 14:**
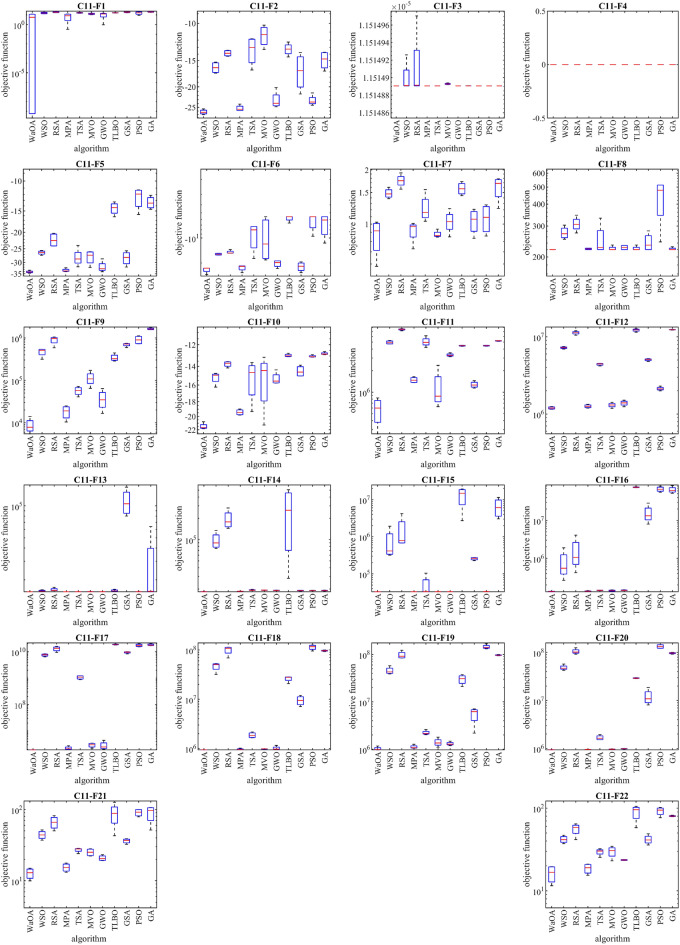
Boxplot diagrams of performance of WaOA and competitor algorithms on the CEC 2011 test suite.

In this subsection, the performance of WaOA in handling real-world applications is challenged on twenty-two constrained optimization problems from the CEC 2011 test suite. This test suite has twenty-two optimization problems, namely: parameter estimation for frequency-modulated (FM) sound waves, the Lennard–Jones potential problem, the bifunctional catalyst blend optimal control problem, optimal control of a nonlinear stirred tank reactor, the Tersoff potential for model Si (B), the Tersoff potential for model Si (C), spread spectrum radar polyphase code design, transmission network expansion planning (TNEP) problem, large-scale transmission pricing problem, circular antenna array design problem, and the ELD problems (which consist of DED instance 1, DED instance 2, ELD Instance 1, ELD Instance 2, ELD Instance 3, ELD Instance 4, ELD Instance 5, hydrothermal scheduling instance 1, hydrothermal scheduling instance 2, and hydrothermal scheduling instance 3), the Messenger spacecraft trajectory optimization problem, and the Cassini 2 spacecraft trajectory optimization problem. Full details and description of the CEC 2011 test suite are available at^64^. The results of employing WaOA and competitor algorithms on these real-world optimization problems are presented in Table 18. The boxplot diagrams obtained from the performance of metaheuristic algorithms in handling CEC 2011 test suite optimization problems are drawn in Fig. 14. Based on the simulation results, WaOA is the first best optimizer to solve all C11–F1 to C11–F22 optimization problems. Based on the simulation results, the proposed WaOA approach has provided better results in most of the optimization problems and has provided superior performance in handling the CEC 2011 test suite in competition with competing algorithms. Also, the results obtained from the statistical analysis for $p$-value show that WaOA has a significant statistical superiority compared to competitor algorithms.

**Table 18:** Evaluation results of the CEC 2011 test suite functions.

	WaOA	WSO	RSA	MPA	TSA	MVO	GWO	TLBO	GSA	PSO	GA
C11–F1											
Mean	5.92E+00	1.60E+01	1.96E+01	7.33E+00	1.66E+01	1.28E+01	1.01E+01	1.66E+01	1.94E+01	1.62E+01	2.08E+01
Best	2.00E−10	1.31E+01	1.72E+01	3.17E−01	1.49E+01	1.07E+01	9.50E−01	1.58E+01	1.67E+01	8.92E+00	1.90E+01
Worst	1.23E+01	1.92E+01	2.26E+01	1.26E+01	1.86E+01	1.53E+01	1.48E+01	1.74E+01	2.15E+01	2.25E+01	2.36E+01
Std	7.20E+00	3.34E+00	2.93E+00	6.06E+00	1.70E+00	2.14E+00	6.60E+00	7.48E−01	2.22E+00	6.16E+00	2.19E+00
Median	5.69E+00	1.58E+01	1.94E+01	8.18E+00	1.64E+01	1.26E+01	1.24E+01	1.67E+01	1.96E+01	1.67E+01	2.04E+01
Rank	1	5	10	2	7	4	3	8	9	6	11
C11–F2											
Mean	− 2.63E+01	− 1.62E+01	− 1.39E+01	− 2.53E+01	− 1.36E+01	− 1.15E+01	− 2.32E+01	− 1.33E+01	− 1.72E+01	− 2.32E+01	− 1.50E+01
Best	− 2.71E+01	− 1.72E+01	− 1.43E+01	− 2.59E+01	− 1.66E+01	− 1.33E+01	− 2.48E+01	− 1.44E+01	− 2.16E+01	− 2.44E+01	− 1.68E+01
Worst	− 2.54E+01	− 1.53E+01	− 1.34E+01	− 2.41E+01	− 1.18E+01	− 1.02E+01	− 2.01E+01	− 1.23E+01	− 1.37E+01	− 2.14E+01	− 1.37E+01
Std	7.39E−01	1.04E+00	4.85E−01	8.62E−01	2.37E+00	1.44E+00	2.22E+00	9.45E−01	3.72E+00	1.38E+00	1.59E+00
Median	− 2.64E+01	− 1.62E+01	− 1.39E+01	− 2.55E+01	− 1.30E+01	− 1.13E+01	− 2.39E+01	− 1.32E+01	− 1.67E+01	− 2.36E+01	− 1.47E+01
Rank	1	6	8	2	9	11	4	10	5	3	7
C11–F3											
Mean	1.15E−05	1.15E−05	1.15E−05	1.15E−05	1.15E−05	1.15E−05	1.15E−05	1.15E−05	1.15E−05	1.15E−05	1.15E−05
Best	1.15E−05	1.15E−05	1.15E−05	1.15E−05	1.15E−05	1.15E−05	1.15E−05	1.15E−05	1.15E−05	1.15E−05	1.15E−05
Worst	1.15E−05	1.15E−05	1.15E−05	1.15E−05	1.15E−05	1.15E−05	1.15E−05	1.15E−05	1.15E−05	1.15E−05	1.15E−05
Std	2.00E−19	1.85E−11	4.17E−11	1.04E−15	1.99E−14	8.31E−13	3.11E−15	6.54E−14	1.68E−19	6.77E−20	2.29E−18
Median	1.15E−05	1.15E−05	1.15E−05	1.15E−05	1.15E−05	1.15E−05	1.15E−05	1.15E−05	1.15E−05	1.15E−05	1.15E−05
Rank	1	10	11	5	7	9	6	8	3	2	4
C11–F4											
Mean	0.00E+00	0.00E+00	0.00E+00	0.00E+00	0.00E+00	0.00E+00	0.00E+00	0.00E+00	0.00E+00	0.00E+00	0.00E+00
Best	0.00E+00	0.00E+00	0.00E+00	0.00E+00	0.00E+00	0.00E+00	0.00E+00	0.00E+00	0.00E+00	0.00E+00	0.00E+00
Worst	0.00E+00	0.00E+00	0.00E+00	0.00E+00	0.00E+00	0.00E+00	0.00E+00	0.00E+00	0.00E+00	0.00E+00	0.00E+00
Std	0.00E+00	0.00E+00	0.00E+00	0.00E+00	0.00E+00	0.00E+00	0.00E+00	0.00E+00	0.00E+00	0.00E+00	0.00E+00
Median	0.00E+00	0.00E+00	0.00E+00	0.00E+00	0.00E+00	0.00E+00	0.00E+00	0.00E+00	0.00E+00	0.00E+00	0.00E+00
Rank	1	1	1	1	1	1	1	1	1	1	1
C11–F5											
Mean	− 3.41E+01	− 2.63E+01	− 2.23E+01	− 3.34E+01	− 2.83E+01	− 2.82E+01	− 3.20E+01	− 1.45E+01	− 2.85E+01	− 1.27E+01	− 1.34E+01
Best	− 3.47E+01	− 2.73E+01	− 2.41E+01	− 3.40E+01	− 3.19E+01	− 3.21E+01	− 3.40E+01	− 1.62E+01	− 3.20E+01	− 1.57E+01	− 1.47E+01
Worst	− 3.34E+01	− 2.55E+01	− 2.03E+01	− 3.23E+01	− 2.39E+01	− 2.60E+01	− 2.86E+01	− 1.33E+01	− 2.57E+01	− 1.13E+01	− 1.21E+01
Std	5.90E−01	7.75E−01	2.17E+00	7.93E−01	3.45E+00	2.99E+00	2.46E+00	1.32E+00	2.88E+00	2.17E+00	1.19E+00
Median	− 3.42E+01	− 2.62E+01	− 2.23E+01	− 3.37E+01	− 2.87E+01	− 2.73E+01	− 3.27E+01	− 1.43E+01	− 2.81E+01	− 1.19E+01	− 1.35E+01
Rank	1	7	8	2	5	6	3	9	4	11	10
C11–F6											
Mean	− 2.41E+01	− 1.57E+01	− 1.48E+01	− 2.29E+01	− 1.02E+01	− 1.19E+01	− 2.04E+01	− 5.83E+00	− 2.23E+01	− 6.55E+00	− 7.32E+00
Best	− 2.74E+01	− 1.60E+01	− 1.52E+01	− 2.60E+01	− 1.76E+01	− 1.83E+01	− 2.32E+01	− 6.63E+00	− 2.60E+01	− 9.54E+00	− 1.15E+01
Worst	− 2.30E+01	− 1.53E+01	− 1.38E+01	− 2.16E+01	− 7.30E+00	− 5.56E+00	− 1.88E+01	− 5.56E+00	− 1.94E+01	− 5.56E+00	− 5.56E+00
Std	2.32E+00	4.16E−01	7.25E−01	2.24E+00	5.20E+00	7.06E+00	2.20E+00	5.62E−01	3.08E+00	2.09E+00	2.99E+00
Median	− 2.30E+01	− 1.57E+01	− 1.52E+01	− 2.19E+01	− 8.01E+00	− 1.18E+01	− 1.97E+01	− 5.56E+00	− 2.18E+01	− 5.56E+00	− 6.09E+00
Rank	1	5	6	2	8	7	4	11	3	10	9
C11–F7											
Mean	8.61E−01	1.48E+00	1.74E+00	9.18E−01	1.23E+00	8.78E−01	1.03E+00	1.58E+00	1.04E+00	1.08E+00	1.59E+00
Best	5.82E−01	1.39E+00	1.56E+00	7.28E−01	1.04E+00	8.46E−01	8.50E−01	1.44E+00	8.34E−01	8.58E−01	1.22E+00
Worst	1.03E+00	1.60E+00	1.93E+00	1.01E+00	1.55E+00	9.37E−01	1.22E+00	1.72E+00	1.21E+00	1.28E+00	1.79E+00
Std	2.12E−01	9.72E−02	1.56E−01	1.35E−01	2.37E−01	4.26E−02	1.59E−01	1.34E−01	1.75E−01	2.14E−01	2.71E−01
Median	9.18E−01	1.47E+00	1.74E+00	9.68E−01	1.16E+00	8.64E−01	1.03E+00	1.57E+00	1.07E+00	1.09E+00	1.68E+00
Rank	1	8	11	3	7	2	4	9	5	6	10
C11–F8											
Mean	2.20E+02	2.74E+02	3.08E+02	2.22E+02	2.51E+02	2.23E+02	2.26E+02	2.23E+02	2.42E+02	4.29E+02	2.22E+02
Best	2.20E+02	2.52E+02	2.74E+02	2.20E+02	2.20E+02	2.20E+02	2.20E+02	2.20E+02	2.20E+02	2.44E+02	2.20E+02
Worst	2.20E+02	3.03E+02	3.46E+02	2.24E+02	3.33E+02	2.34E+02	2.32E+02	2.34E+02	2.81E+02	5.13E+02	2.28E+02
Std	0.00E+00	2.36E+01	3.09E+01	2.49E+00	5.74E+01	7.17E+00	7.46E+00	7.17E+00	3.06E+01	1.34E+02	4.38E+00
Median	2.20E+02	2.71E+02	3.07E+02	2.22E+02	2.26E+02	2.20E+02	2.26E+02	2.20E+02	2.33E+02	4.79E+02	2.20E+02
Rank	1	8	9	2	7	4	5	4	6	10	3
C11–F9											
Mean	8.79E+03	4.68E+05	8.91E+05	1.83E+04	5.69E+04	1.13E+05	3.75E+04	3.44E+05	6.91E+05	9.08E+05	1.63E+06
Best	5.46E+03	3.14E+05	5.83E+05	1.04E+04	4.07E+04	6.46E+04	1.66E+04	2.85E+05	5.93E+05	7.29E+05	1.56E+06
Worst	1.40E+04	5.37E+05	1.04E+06	2.49E+04	7.18E+04	1.72E+05	6.39E+04	4.42E+05	7.44E+05	1.11E+06	1.72E+06
Std	3.89E+03	1.11E+05	2.20E+05	6.98E+03	1.41E+04	4.64E+04	2.10E+04	7.27E+04	7.07E+04	2.15E+05	8.42E+04
Median	7.83E+03	5.11E+05	9.68E+05	1.90E+04	5.76E+04	1.08E+05	3.47E+04	3.25E+05	7.14E+05	8.96E+05	1.61E+06
Rank	1	7	9	2	4	5	3	6	8	10	11
C11–F10											
Mean	− 2.15E+01	− 1.52E+01	− 1.38E+01	− 1.94E+01	− 1.55E+01	− 1.58E+01	− 1.53E+01	− 1.30E+01	− 1.45E+01	− 1.30E+01	− 1.28E+01
Best	− 2.18E+01	− 1.62E+01	− 1.42E+01	− 1.98E+01	− 1.93E+01	− 2.13E+01	− 1.58E+01	− 1.31E+01	− 1.50E+01	− 1.31E+01	− 1.29E+01
Worst	− 2.08E+01	− 1.47E+01	− 1.35E+01	− 1.90E+01	− 1.36E+01	− 1.31E+01	− 1.44E+01	− 1.28E+01	− 1.39E+01	− 1.29E+01	− 1.26E+01
Std	4.99E−01	7.32E−01	2.97E−01	4.17E−01	2.70E+00	3.88E+00	6.90E−01	1.46E−01	5.93E−01	1.09E−01	1.29E−01
Median	− 2.17E+01	− 1.49E+01	− 1.37E+01	− 1.95E+01	− 1.46E+01	− 1.44E+01	− 1.56E+01	− 1.30E+01	− 1.46E+01	− 1.31E+01	− 1.28E+01
Rank	1	6	8	2	4	3	5	10	7	9	11
C11–F11											
Mean	5.72E+05	4.95E+06	7.51E+06	1.48E+06	5.07E+06	1.19E+06	3.30E+06	4.45E+06	1.27E+06	4.46E+06	5.22E+06
Best	2.61E+05	4.71E+06	7.21E+06	1.36E+06	4.22E+06	6.27E+05	3.13E+06	4.38E+06	1.14E+06	4.40E+06	5.13E+06
Worst	8.29E+05	5.28E+06	7.71E+06	1.64E+06	6.13E+06	2.37E+06	3.55E+06	4.51E+06	1.44E+06	4.51E+06	5.26E+06
Std	2.61E+05	2.93E+05	2.23E+05	1.45E+05	8.29E+05	8.37E+05	1.87E+05	6.31E+04	1.38E+05	5.92E+04	6.50E+04
Median	5.99E+05	4.90E+06	7.56E+06	1.47E+06	4.96E+06	8.79E+05	3.26E+06	4.46E+06	1.26E+06	4.47E+06	5.24E+06
Rank	1	8	11	4	9	2	5	6	3	7	10
C11–F12											
Mean	1.20E+06	7.22E+06	1.13E+07	1.26E+06	4.40E+06	1.31E+06	1.39E+06	1.22E+07	5.04E+06	2.13E+06	1.23E+07
Best	1.16E+06	6.92E+06	1.05E+07	1.19E+06	4.19E+06	1.18E+06	1.24E+06	1.15E+07	4.80E+06	2.00E+06	1.22E+07
Worst	1.25E+06	7.48E+06	1.20E+07	1.34E+06	4.52E+06	1.42E+06	1.51E+06	1.27E+07	5.21E+06	2.30E+06	1.24E+07
Std	4.72E+04	2.44E+05	6.44E+05	6.68E+04	1.62E+05	1.02E+05	1.17E+05	5.45E+05	1.83E+05	1.31E+05	9.84E+04
Median	1.20E+06	7.23E+06	1.13E+07	1.26E+06	4.46E+06	1.31E+06	1.40E+06	1.23E+07	5.07E+06	2.12E+06	1.23E+07
Rank	1	8	9	2	6	3	4	10	7	5	11
C11–F13											
Mean	1.54E+04	1.58E+04	1.62E+04	1.55E+04	1.55E+04	1.55E+04	1.55E+04	1.59E+04	1.10E+05	1.55E+04	2.76E+04
Best	1.54E+04	1.56E+04	1.58E+04	1.55E+04	1.55E+04	1.55E+04	1.55E+04	1.56E+04	8.02E+04	1.55E+04	1.55E+04
Worst	1.54E+04	1.62E+04	1.70E+04	1.55E+04	1.55E+04	1.55E+04	1.55E+04	1.63E+04	1.50E+05	1.55E+04	6.39E+04
Std	9.09E−03	2.66E+02	6.12E+02	2.46E+00	9.80E+00	2.40E+01	7.37E+00	3.46E+02	3.32E+04	2.17E+01	2.54E+04
Median	1.54E+04	1.57E+04	1.59E+04	1.55E+04	1.55E+04	1.55E+04	1.55E+04	1.57E+04	1.05E+05	1.55E+04	1.56E+04
Rank	1	7	9	2	3	6	5	8	11	4	10
C11–F14											
Mean	1.83E+04	9.72E+04	1.95E+05	1.86E+04	1.93E+04	1.92E+04	1.91E+04	2.63E+05	1.90E+04	1.90E+04	1.90E+04
Best	1.82E+04	7.46E+04	1.44E+05	1.85E+04	1.91E+04	1.91E+04	1.89E+04	2.83E+04	1.87E+04	1.89E+04	1.87E+04
Worst	1.84E+04	1.35E+05	2.79E+05	1.86E+04	1.98E+04	1.93E+04	1.92E+04	5.05E+05	1.91E+04	1.91E+04	1.92E+04
Std	7.16E+01	2.82E+04	6.36E+04	6.95E+01	3.20E+02	7.17E+01	1.30E+02	2.41E+05	1.90E+02	1.17E+02	2.06E+02
Median	1.83E+04	8.97E+04	1.78E+05	1.86E+04	1.92E+04	1.93E+04	1.91E+04	2.60E+05	1.90E+04	1.90E+04	1.90E+04
Rank	1	9	10	2	8	7	6	11	3	5	4
C11–F15											
Mean	3.29E+04	7.66E+05	1.61E+06	3.29E+04	5.10E+04	3.31E+04	3.30E+04	1.29E+07	2.57E+05	3.32E+04	6.66E+06
Best	3.28E+04	3.19E+05	6.76E+05	3.29E+04	3.30E+04	3.30E+04	3.30E+04	2.71E+06	2.28E+05	3.32E+04	3.03E+06
Worst	3.30E+04	1.92E+06	4.19E+06	3.30E+04	1.05E+05	3.31E+04	3.31E+04	1.93E+07	2.76E+05	3.32E+04	1.14E+07
Std	7.69E+01	8.11E+05	1.81E+06	6.60E+01	3.77E+04	6.45E+01	5.09E+01	7.92E+06	2.38E+04	1.74E+01	4.03E+06
Median	3.29E+04	4.14E+05	7.85E+05	3.29E+04	3.31E+04	3.31E+04	3.30E+04	1.49E+07	2.61E+05	3.32E+04	6.09E+06
Rank	1	8	9	2	6	4	3	11	7	5	10
C11–F16											
Mean	1.34E+05	8.14E+05	1.66E+06	1.37E+05	1.43E+05	1.40E+05	1.44E+05	7.45E+07	1.57E+07	6.67E+07	6.40E+07
Best	1.31E+05	2.64E+05	4.19E+05	1.35E+05	1.41E+05	1.33E+05	1.42E+05	7.26E+07	7.99E+06	5.52E+07	5.18E+07
Worst	1.36E+05	1.89E+06	4.08E+06	1.41E+05	1.45E+05	1.47E+05	1.49E+05	7.67E+07	2.84E+07	7.97E+07	8.19E+07
Std	2.39E+03	7.70E+05	1.73E+06	2.64E+03	2.09E+03	6.15E+03	3.58E+03	1.78E+06	9.28E+06	1.11E+07	1.35E+07
Median	1.33E+05	5.49E+05	1.06E+06	1.36E+05	1.44E+05	1.40E+05	1.42E+05	7.44E+07	1.32E+07	6.60E+07	6.12E+07
Rank	1	6	7	2	4	3	5	11	8	10	9
C11–F17											
Mean	1.93E+06	7.51E+09	1.30E+10	2.23E+06	1.07E+09	2.92E+06	2.84E+06	1.87E+10	9.40E+09	1.75E+10	1.83E+10
Best	1.92E+06	6.40E+09	9.34E+09	1.95E+06	8.86E+08	2.24E+06	2.02E+06	1.80E+10	8.27E+09	1.54E+10	1.71E+10
Worst	1.94E+06	8.33E+09	1.59E+10	2.75E+06	1.23E+09	3.45E+06	4.40E+06	1.95E+10	9.96E+09	2.02E+10	2.07E+10
Std	1.20E+04	8.96E+08	2.96E+09	3.75E+05	1.85E+08	5.86E+05	1.13E+06	6.63E+08	8.05E+08	2.26E+09	1.70E+09
Median	1.92E+06	7.66E+09	1.34E+10	2.11E+06	1.09E+09	3.00E+06	2.48E+06	1.86E+10	9.68E+09	1.71E+10	1.77E+10
Rank	1	6	8	2	5	4	3	11	7	9	10
C11–F18											
Mean	9.42E+05	4.62E+07	9.94E+07	9.67E+05	1.87E+06	9.81E+05	1.02E+06	2.61E+07	9.48E+06	1.13E+08	9.61E+07
Best	9.38E+05	3.18E+07	6.87E+07	9.48E+05	1.66E+06	9.61E+05	9.63E+05	2.07E+07	7.11E+06	9.50E+07	9.26E+07
Worst	9.45E+05	5.26E+07	1.13E+08	1.02E+06	2.16E+06	9.89E+05	1.16E+06	2.83E+07	1.19E+07	1.26E+08	9.97E+07
Std	2.77E+03	1.02E+07	2.20E+07	3.46E+04	2.54E+05	1.41E+04	1.00E+05	3.79E+06	2.26E+06	1.44E+07	3.03E+06
Median	9.43E+05	5.03E+07	1.08E+08	9.52E+05	1.84E+06	9.86E+05	9.72E+05	2.78E+07	9.44E+06	1.16E+08	9.61E+07
Rank	1	8	10	2	5	3	4	7	6	11	9
C11–F19											
Mean	1.03E+06	4.55E+07	9.73E+07	1.12E+06	2.23E+06	1.40E+06	1.31E+06	3.00E+07	5.42E+06	1.45E+08	9.65E+07
Best	9.68E+05	3.89E+07	8.41E+07	1.05E+06	2.02E+06	1.10E+06	1.19E+06	2.10E+07	2.19E+06	1.31E+08	9.41E+07
Worst	1.17E+06	5.78E+07	1.22E+08	1.27E+06	2.59E+06	1.80E+06	1.48E+06	3.74E+07	7.06E+06	1.67E+08	9.93E+07
Std	9.97E+04	8.99E+06	1.87E+07	1.08E+05	2.63E+05	3.06E+05	1.30E+05	7.43E+06	2.32E+06	1.64E+07	2.28E+06
Median	9.83E+05	4.27E+07	9.15E+07	1.08E+06	2.15E+06	1.36E+06	1.28E+06	3.08E+07	6.21E+06	1.40E+08	9.62E+07
Rank	1	8	10	2	5	4	3	7	6	11	9
C11–F20											
Mean	9.41E+05	4.84E+07	1.05E+08	9.57E+05	1.68E+06	9.68E+05	9.89E+05	2.91E+07	1.21E+07	1.34E+08	9.67E+07
Best	9.36E+05	4.26E+07	9.20E+07	9.55E+05	1.53E+06	9.60E+05	9.73E+05	2.85E+07	8.10E+06	1.22E+08	9.21E+07
Worst	9.47E+05	5.73E+07	1.25E+08	9.58E+05	1.94E+06	9.77E+05	1.00E+06	2.98E+07	1.87E+07	1.45E+08	1.00E+08
Std	5.01E+03	6.57E+06	1.48E+07	1.30E+03	2.05E+05	7.68E+03	1.34E+04	5.78E+05	4.85E+06	1.34E+07	3.64E+06
Median	9.41E+05	4.68E+07	1.02E+08	9.58E+05	1.63E+06	9.67E+05	9.91E+05	2.91E+07	1.08E+07	1.34E+08	9.73E+07
Rank	1	8	10	2	5	3	4	7	6	11	9
C11–F21											
Mean	1.27E+01	4.42E+01	6.62E+01	1.54E+01	2.71E+01	2.51E+01	2.08E+01	8.65E+01	3.62E+01	9.07E+01	8.81E+01
Best	9.97E+00	3.71E+01	5.01E+01	1.31E+01	2.41E+01	2.23E+01	1.91E+01	4.28E+01	3.25E+01	7.89E+01	5.16E+01
Worst	1.50E+01	5.17E+01	8.21E+01	1.77E+01	2.84E+01	2.81E+01	2.31E+01	1.26E+02	3.89E+01	1.00E+02	1.07E+02
Std	2.41E+00	6.61E+00	1.48E+01	2.21E+00	2.10E+00	3.18E+00	1.96E+00	3.59E+01	2.96E+00	1.13E+01	2.70E+01
Median	1.30E+01	4.39E+01	6.62E+01	1.54E+01	2.79E+01	2.51E+01	2.05E+01	8.84E+01	3.67E+01	9.18E+01	9.70E+01
Rank	1	7	8	2	5	4	3	9	6	11	10
C11–F22											
Mean	1.61E+01	4.19E+01	5.58E+01	1.86E+01	2.96E+01	2.97E+01	2.36E+01	8.86E+01	4.18E+01	9.20E+01	8.02E+01
Best	1.15E+01	3.73E+01	4.17E+01	1.54E+01	2.54E+01	2.30E+01	2.32E+01	5.79E+01	3.58E+01	7.68E+01	7.87E+01
Worst	1.96E+01	4.70E+01	6.44E+01	2.10E+01	3.22E+01	3.45E+01	2.39E+01	1.05E+02	4.89E+01	1.02E+02	8.21E+01
Std	4.20E+00	4.48E+00	1.04E+01	2.81E+00	3.06E+00	5.13E+00	3.94E−01	2.22E+01	5.75E+00	1.17E+01	1.51E+00
Median	1.67E+01	4.16E+01	5.86E+01	1.90E+01	3.03E+01	3.06E+01	2.36E+01	9.58E+01	4.11E+01	9.47E+01	8.01E+01
Rank	1	7	8	2	4	5	3	10	6	11	9
Sum rank	22	153	190	49	124	100	86	184	127	168	187
Mean rank	1.00E+00	6.95E+00	8.64E+00	2.23E+00	5.64E+00	4.55E+00	3.91E+00	8.36E+00	5.77E+00	7.64E+00	8.50E+00
Total rank	1	7	11	2	5	4	3	9	6	8	10
*p*-value		1.71E−15	1.71E−15	7.1E−15	3.66E−15	3.99E−12	7.1E−15	5.36E−15	8.52E−15	2.54E−15	5.36E−15

## Conclusions and future works

In this study, a new bio-inspired metaheuristic algorithm called the Walrus Optimization Algorithm (WaOA) was developed based on the natural behaviors of walruses. Feeding, escaping, fighting predators, and migrating are the primary sources of inspiration used in the design of WaOA. Therefore, the WaOA theory was explained, and its mathematical modeling was presented in three phases: (i) feeding strategy, (ii) migration, and (iii) escaping and fighting against predators. Sixty-eight standard benchmark functions of various types of unimodal, multimodal, the CEC 2015 test suite, and the CEC 2017 test suite, were employed to analyze WaOA performance in providing solutions. The information on these test functions is specified in the Appendix and Tables A1 to A5. The optimization results of unimodal functions showed the high ability of WaOA exploitation in local search to converge towards global optimal. The optimization results of multimodal functions indicated the high ability of WaOA exploration in global search and not to be caught in locally optimal solutions. WaOA’s performance results were compared with the ten well-known metaheuristic algorithms. The simulation and comparison results showed that the proposed WaOA approach has a high ability to balance exploration and exploitation and is much superior and more competitive against ten competitor metaheuristic algorithms. In addition, the results of the WaOA implementation in addressing the four design issues and twenty-two real-world optimization problems from the CEC 2011 test suite demonstrates the effectiveness of the proposed approach in real-world applications.

Although it was observed that WaOA had provided superior results in most of the benchmark functions, the proposed approach has some limitations. The first limitation facing all metaheuristic algorithms is that it is always possible to design newer algorithms that can provide better results than existing algorithms. The second limitation of WaOA is that the proposed method may fail in some optimization applications. The third limitation of WaOA is that the nature of random search in this algorithm leads to the fact that there is no guarantee of achieving the global optimum. Moreover, the authors do not claim that the proposed WaOA approach is the best optimizer for all possible optimization tasks. This fact, of course, cannot be said about any optimizer due to the validity of the NFL theorem.

The authors offer several study directions for future research, including designing the multi-objective version of WaOA and the binary version of WaOA. In addition, the use of WaOA in solving optimization problems in real-world applications is a possible line for further research.

## Supplementary Information

Supplementary Tables.

## Data Availability

All data generated or analyzed during this study are included directly in the text of this submitted manuscript. There are no additional external files with datasets.
