# Supplementary material for: A new bio-inspired metaheuristic algorithm for solving optimization problems based on walruses behavior
Source: Sci Rep. 2023 May 31;13:8775. doi: 10.1038/s41598-023-35863-5 (PMC10232466; doi:10.1038/s41598-023-35863-5)
Supplement: Supplementary file 1 — Supplementary Tables. [file 41598_2023_35863_MOESM1_ESM.docx]

**Appendix**

The appendix contains complete information on the objective functions used in the simulation section. Table A1 contains the definitions, ranges, dimensions, and minimum values of the seven commonly used unimodal objective functions. Table A2 is devoted to a summary of information on six high-dimensional multimodal objective functions, and Table A3 contains a complete overview of ten fixed-dimensional multimodal objective functions.

Table A5 and A6 contains the definitions, ranges, dimensions, and minimum values of the CEC-2015 and CEC-2017 test suite, respectively.

| **Objective Function** | **Range** | ***Dim*** | $\boldsymbol{F}_{\boldsymbol{min}}$ |
| --- | --- | --- | --- |
| $F_{1}(X)=\sum_{i=1}^{m} x_{i}^{2}$ | $\left[ -100,100 \right]$ | *30* | *0* |
| $F_{2}\left( X \right)=\sum_{i=1}^{m} \left\vert x_{i} \right\vert+ \prod_{i=1}^{m} \left\vert x_{i} \right\vert$ | $\left[ -10,10 \right]$ | 30 | 0 |
| $F_{3}\left( X \right)=\sum_{i=1}^{m} \left( \sum_{j=1}^{i} x_{i} \right)^{2}$ | $\left[ -100,100 \right]$ | 30 | 0 |
| $F_{4}\left( X \right)=max\left\{ \left\vert x_{i} \right\vert\right\}, 1\leq i\leq m$, | $\left[ -100,100 \right]$ | 30 | 0 |
| $F_{5}\left( X \right)=\sum_{i=1}^{m-1} \left[ 100\left( x_{i+1}-x_{i}^{2} \right)^{2}+\left( x_{i}-1 \right)^{2}) \right]$ | $\left[ -30,30 \right]$ | 30 | 0 |
| $F_{6}\left( X \right)=\sum_{i=1}^{m} \left\lfloor x_{i}+0.5 \right\rfloor^{2}$ | $\left[ -100,100 \right]$ | 30 | 0 |
| $F_{7}\left( X \right)=\sum_{i=1}^{m} {ix}_{i}^{4}+r,\mathrm{where} r is a random real number from the interval [0,1]$ | $\left[ -1.28,1.28 \right]$ | 30 | 0 |

Table A1.  Unimodal objective functions.

| **Objective Function** | **Range** | ***Dim*** | $\boldsymbol{F}_{\boldsymbol{min}}$ |
| --- | --- | --- | --- |
| $F_{8}\left( X \right)=\sum_{i=1}^{m} -x_{i}\sin\left( \sqrt{\left\vert x_{i} \right\vert} \right)$ | $\left[ -500,500 \right]$ | 30 | −12569 |
| $F_{9}\left( X \right)=\sum_{i=1}^{m} \left[ x_{i}^{2}-10\cos\left( 2\pi x_{i} \right)+10 \right]$ | $\left[ -5.12,5.12 \right]$ | 30 | 0 |
| $F_{10}\left( X \right)=-20\exp\left( -0.2\sqrt{\frac{1}{m}\sum_{i=1}^{m} x_{i}^{2}} \right)-\exp\left( \frac{1}{m}\sum_{i=1}^{m} \cos\left( 2\pi x_{i} \right) \right)+20+e$ | $\left[ -32,32 \right]$ | 30 | 0 |
| $F_{11}\left( X \right)=\frac{1}{4000}\sum_{i=1}^{m} x_{i}^{2}- \prod_{i=1}^{m} cos\left( \frac{x_{i}}{\sqrt{i}} \right)+1$ | $\left[ -600,600 \right]$ | 30 | 0 |
| $F_{12}\left( X \right)=\frac{\pi}{m} \left\{ 10\sin\left( \pi y_{1} \right)+\sum_{i=1}^{m} \left( y_{i}-1 \right)^{2}\left[ 1+10\sin^{2} \left( \pi y_{i+1} \right) \right]+\left( y_{n}-1 \right)^{2} \right\}+\sum_{i=1}^{m} u\left( x_{i},10,100,4 \right)$, where  $y_{i}= 1+\frac{x_{i}+1}{4}$, $u\left( x_{i},a,i,n \right)= \left\{ \begin{matrix} k\left( x_{i}-a \right)^{n}, & x_{i}>a; \\ 0, & -a\leq x_{i} \leq a; \\ k\left( {-x}_{i}-a \right)^{n}, & x_{i}<-a, \end{matrix} \right.$ | $\left[ -50,50 \right]$ | 30 | 0 |
| $F_{13}\left( X \right)=0.1\left\{ \sin^{2} \left( 3\pi x_{1} \right)+ \sum_{i=1}^{m} \left( x_{i}-1 \right)^{2}\left[ 1+\sin^{2} \left( 3\pi x_{i}+1 \right) \right]+\left( x_{n}-1 \right)^{2}\left[ 1+\sin^{2} \left( 2\pi x_{m} \right) \right] \right\}+\sum_{i=1}^{m} u\left( x_{i},5,100,4 \right),$ where  $u\left( x_{i},a,i,n \right)= \left\{ \begin{matrix} k\left( x_{i}-a \right)^{n}, & x_{i}>a; \\ 0, & -a\leq x_{i} \leq a; \\ k\left( {-x}_{i}-a \right)^{n}, & x_{i}<-a. \end{matrix} \right.$ | $\left[ -50,50 \right]$ | 30 | 0 |

Table A2.  High-dimensional multimodal objective functions.

| **Objective Function** | **Range** | ***Dim*** | $\boldsymbol{F}_{\boldsymbol{min}}$ |
| --- | --- | --- | --- |
| $F_{14}\left( X \right)=\left( \frac{1}{500}+\sum_{j=1}^{25} \frac{1}{j+\sum_{i=1}^{2} \left( x_{i}-a_{ij} \right)^{6}} \right)^{-1}$ | $\left[ -65.53,65.53 \right]$ | 2 | 0.998 |
| $F_{15}\left( X \right)=\sum_{i=1}^{11} \left[ a_{i}-\frac{x_{1}\left( b_{i}^{2}+b_{i}x_{2} \right)}{b_{i}^{2}+b_{i}x_{3}+x_{4}} \right]^{2}$ | $\left[ -5,5 \right]$ | 4 | 0.00030 |
| $F_{16}\left( X \right)=4x_{1}^{2}-2.1\cdot x_{1}^{4}+\frac{1}{3}x_{1}^{6}+x_{1}x_{2}-4x_{2}^{2}+4x_{2}^{4}$ | $\left[ -5,5 \right]$ | 2 | −1.0316 |
| $F_{17}\left( X \right)=\left( x_{2}-\frac{5.1}{4\pi^{2}}x_{1}^{2}+\frac{5}{\pi}x_{1}-6 \right)^{2}+10\left( 1-\frac{1}{8\pi} \right)cosx_{1}+10$ | [-5,10]$\times$[0,15] | 2 | 0.398 |
| $F_{18}\left( X \right)=\left[ 1+\left( x_{1}+x_{2}+1 \right)^{2}\left( 19-14x_{1}+3x_{1}^{2}-14x_{2}+6x_{1}x_{2}+3x_{2}^{2} \right) \right]$ $\left[ 30+\left( 2x_{1}-3x_{2} \right)^{2}\left( 18-32x_{1}+12x_{1}^{2}+48x_{2}-36x_{1}x_{2}+27x_{2}^{2} \right) \right]$ | $\left[ -5,5 \right]$ | 2 | 3 |
| $F_{19}\left( X \right)=-\sum_{i=1}^{4} c_{i}\exp\left( -\sum_{j=1}^{3} a_{ij}\left( x_{j}-p_{ij} \right)^{2} \right)$ | $\left[ 0,1 \right]$ | 3 | −3.86 |
| $F_{20}\left( X \right)=-\sum_{i=1}^{4} c_{i}\exp\left( -\sum_{j=1}^{6} a_{ij}\left( x_{j}-p_{ij} \right)^{2} \right)$ | $\left[ 0,1 \right]$ | 6 | −3.22 |
| $F_{21}\left( X \right)=-\sum_{i=1}^{5} \left[ \left( X-a_{i} \right)\cdot\left( X-a_{i} \right)^{T}+6c_{i} \right]^{-1}$ | $\left[ 0,10 \right]$ | 4 | −10.1532 |
| $F_{22}\left( X \right)=-\sum_{i=1}^{7} \left[ \left( X-a_{i} \right)\cdot\left( X-a_{i} \right)^{T}+6c_{i} \right]^{-1}$ | $\left[ 0,10 \right]$ | 4 | −10.4029 |
| $F_{23}\left( X \right)=-\sum_{i=1}^{10} \left[ \left( X-a_{i} \right){\cdot\left( X-a_{i} \right)}^{T}+6c_{i} \right]^{-1}$ | $\left[ 0,10 \right]$ | 4 | −10.5364 |

Table A3. Fixed-dimensional multimodal objective functions.

|  | Functions | Related basic functions | Dim | $F_{min}$ |
| --- | --- | --- | --- | --- |
| CEC1 | Rotated Bent Cigar Function | Bent Cigar Function | 30 | 100 |
| CEC2 | Rotated Discus Function | Discus Function | 30 | 200 |
| CEC3 | Shifted and Rotated Weierstrass Function | Weierstrass Function | 30 | 300 |
| CEC4 | Shifted and Rotated Schwefel’s Function | Schwefel’s Function | 30 | 400 |
| CEC5 | Rotated Katsuura | Katsuura Function | 30 | 500 |
| CEC6 | Shifted and Rotated HappyCat Function | HappyCat Function | 30 | 600 |
| CEC7 | Shifted and Rotated HGBat Function | HGBat Function | 30 | 700 |
| CEC8 | Shifted and Rotated Expanded Griewank’s plus Rosenbrock’s Function | Griewank’s Function  Rosenbrock’s Function | 30 | 800 |
| CEC9 | Shifted and Rotated Expanded Scaffer’s F6 Function | Expanded Scaffer’s F6 Function | 30 | 900 |
| CEC10 | Hybrid Function 1 (𝑁 = 3) | Schwefel’s Function  Rastrigin’s Function  High Conditioned Elliptic Function | 30 | 1000 |
| CEC11 | Hybrid Function 2 (𝑁 = 4) | Griewank’s Function  Weierstrass Function  Rosenbrock’s Function  Scaffer’s F6 Function | 30 | 1100 |
| CEC12 | Hybrid Function 3 (𝑁 = 5) | Katsuura Function  HappyCat Function  Expanded Griewank’s plus Rosenbrock’s Function  Schwefel’s Function  Ackley’s Function | 30 | 1200 |
| CEC13 | Composition Function 1 (𝑁 = 5) | Rosenbrock’s Function  High Conditioned Elliptic Function  Bent Cigar Function  Discus Function  High Conditioned Elliptic Function | 30 | 1300 |
| CEC14 | Composition Function 2 (𝑁 = 3) | Schwefel’s Function  Rastrigin’s Function  High Conditioned Elliptic Function | 30 | 1400 |
| CEC15 | Composition Function 3 (𝑁 = 5) | HGBat Function  Rastrigin’s Function  Schwefel’s Function  Weierstrass Function  High Conditioned Elliptic Function | 30 | 1500 |

Table A4.  The IEEE CEC-2015 benchmark test functions.

|  | Functions | | | $F_{min}$ |
| --- | --- | --- | --- | --- |
| C17-F1 | | Shifted and Rotated Bent Cigar Function | | 100 |
| C17-F2 | | Shifted and Rotated Sum of Different Power Function | | 200 |
| C17-F3 | | Shifted and Rotated Zakharov Function | | 300 |
| C17-F4 | | Shifted and Rotated Rosenbrock’s Function | | 400 |
| C17-F5 | | Shifted and Rotated Rastrigin’s Function | | 500 |
| C17-F6 | | Shifted and Rotated Expanded Scaffer’s Function | | 600 |
| C17-F7 | | Shifted and Rotated Lunacek Bi_Rastrigin Function | | 700 |
| C17-F8 | | Shifted and Rotated Non-Continuous Rastrigin’s Function | | 800 |
| C17-F9 | | Shifted and Rotated Levy Function | | 900 |
| C17-F10 | | Shifted and Rotated Schwefel’s Function | | 1000 |
| C17-F11 | | Hybrid Function 1 (𝑁 = 3) | | 1100 |
| C17-F12 | | Hybrid Function 2 (𝑁 = 3) | | 1200 |
| C17-F13 | | Hybrid Function 3 (𝑁 = 3) | | 1300 |
| C17-F14 | | Hybrid Function 4 (𝑁 = 4) | | 1400 |
| C17-F15 | | Hybrid Function 5 (𝑁 = 4) | | 1500 |
| C17-F16 | | | Hybrid Function 6 (𝑁 = 4) | 1600 |
| C17-F17 | | | Hybrid Function 6 (𝑁 = 5) | 1700 |
| C17-F18 | | | Hybrid Function 6 (𝑁 = 5) | 1800 |
| C17-F19 | | | Hybrid Function 6 (𝑁 = 5) | 1900 |
| C17-F20 | | | Hybrid Function 6 (𝑁 = 6) | 2000 |
| C17-F21 | | | Composition Function 1 (𝑁 = 3) | 2100 |
| C17-F22 | | | Composition Function 2 (𝑁 = 3) | 2200 |
| C17-F23 | | | Composition Function 3 (𝑁 = 4) | 2300 |
| C17-F24 | | | Composition Function 4 (𝑁 = 4) | 2400 |
| C17-F25 | | | Composition Function 5 (𝑁 = 5) | 2500 |
| C17-F26 | | | Composition Function 6 (𝑁 = 5) | 2600 |
| C17-F27 | | | Composition Function 7 (𝑁 = 6) | 2700 |
| C17-F28 | | | Composition Function 8 (𝑁 = 6) | 2800 |
| C17-F29 | | | Composition Function 9 (𝑁 = 3) | 2900 |
| C17-F30 | | | Composition Function 10 (𝑁 = 3) | 3000 |

Table A5.  The IEEE CEC-2017 benchmark test functions.
